# Sequencing of Polyclonal Antibodies by Integrating Intact Mass, Middle–Down, and *De Novo* Bottom–Up Mass Spectrometry

**DOI:** 10.1016/j.mcpro.2025.101088

**Published:** 2025-10-13

**Authors:** Lei Xin, Wenting Li, Shuyang Zhang, Ngoc Hieu Tran, Zheng Chen, Jun Ma, Chao Peng, Ailee Aihemaiti, Kyle Hoffman, Xiyue Zhang, Weiping Sun, Linting Li, Zihao Wang, Ming Li, Baozhen Shan

**Affiliations:** 1 Bioinformatics Solutions Inc., Waterloo, Ontario, Canada; 2 BaizhenBio Inc., Wuhan, China; 3 Central China Institute of Artificial Intelligence, Zhengzhou, China; 4 David R. Cheriton School of Computer Science, University of Waterloo, Ontario, Canada

**Keywords:** polyclonal antibody sequencing, *de novo* sequencing, intact mass, middle–down, bottom–up

## Abstract

Polyclonal antibodies (pAbs) represent nature's approach to robust immunity, targeting multiple sites on pathogens, but their complex mixtures have remained largely unsequenceable, limiting their therapeutic potential. While monoclonal antibodies (mAbs) dominate therapeutics because of their reproducibility, pAbs offer superior resilience against viral mutations and broader target recognition. Current pAb sequencing attempts have shown limitations, requiring germline databases or B-cell sequencing. Due to the highly variable nature of antibodies, as well as the possibility of unavailable B cells, there is a need for a purely mass spectrometry– and *de novo* sequencing–based solution. Here, we present PolySeq.AI, an automated *de novo* workflow that combines bottom–up, middle–down, and intact mass analysis, to accurately sequence pAb samples without relying on external databases. PolySeq.AI achieved >99% sequencing accuracy across all tested samples, including an mAb mixture from the HB-95 cell line and a mixture of four mAbs, with complete bottom–up coverage and strong middle–down fragment support. Importantly, recombinant antibodies produced from our *de novo* sequences of HB-95 antibodies retained full binding capabilities to human leukocyte antigen-I complexes, confirming the accuracy and efficacy of our pAb *de novo* sequencing workflow.

## Highlights

• PolySeq.AI integrates intact, middle–down, and bottom–up mass spectrometry.
• Polyclonal antibody *de novo* sequencing with targeted middle–down and bottom–up methods.
• Antibody forms characterization using intact mass deconvolution.
• HB-95 cell line–generated monoclonal antibody is sequenced and validated.
• Mixture of four monoclonal antibodies sequenced and characterized with 99% accuracy.

## Graphical Abstract

**Figure:**
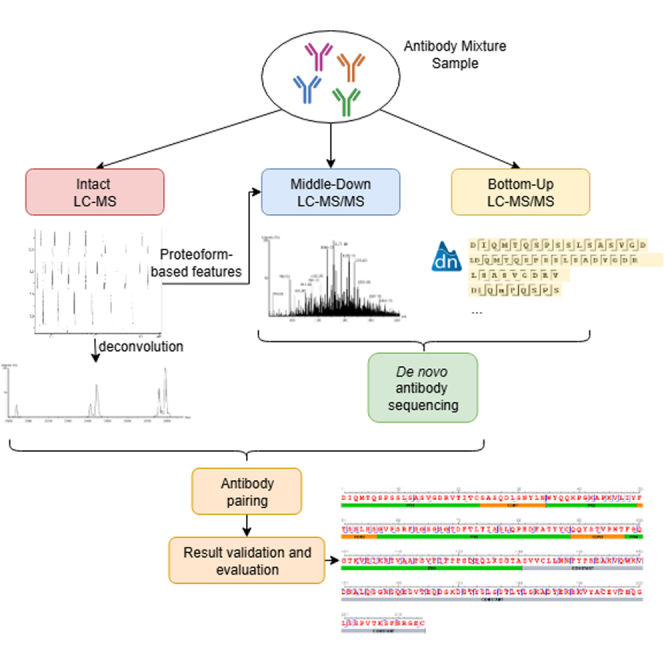

## In Brief

Integrating intact, middle–down, and bottom–up mass spectrometry enables antibody mixture sequencing. PolySeq.AI is a fully automated and *de novo* workflow with a novel peptide assembly algorithm using targeted middle–down fragments and bottom–up peptides. From experiments of the HB-95 cell line–generated monoclonal antibody and a mixture of four monoclonal antibodies, PolySeq.AI successfully sequences antibodies in the mixtures with 99% to 100% accuracy, demonstrating potential applications in recombinant polyclonal antibody production for therapeutics and antigen discovery.

Produced by the adaptive immune system, antibodies are highly functional proteins that can specifically recognize and neutralize harmful antigens, such as those from bacteria or viruses. Due to their ability to accurately target specific epitopes, antibodies are widely used in biological and medical research. Since the US Food and Drug Administration approved the first monoclonal antibody (mAb) Muromonab-CD3 in 1986, the development of diagnostic and therapeutic antibodies has advanced significantly, driving the market’s rapid growth to hundreds of billions of dollars (1, 2, 3). New generations of therapeutic antibodies continue to be developed with greater efficacy and safety, offering treatments for a wide range of diseases, including cancer, autoimmune, metabolic, and infectious diseases (4, 5). Recombinant antibodies have become the preferred choice for therapeutic use because of their high reproducibility and versatility (6). In most cases, genetic engineering is applied to an identified antibody, and the optimized antibody sequence is then produced through bacterial or mammalian cell expression systems. Phage and yeast display have also shown potential in producing recombinant antibodies (7, 8). Therefore, accurately identifying and sequencing natural antibodies is essential for producing recombinant antibodies with high binding affinity and specificity.

The immune system, in response to an antigen, produces B cells, which generate natural antibodies to target and neutralize the antigen. Due to the genetic variation of the B cells, the produced antibodies are called “polyclonal antibodies” (pAbs) (9). On the contrary, mAbs are produced by the hybridoma technology, where one antibody-producing B cell is fused with an immortal cancerous myeloma cell, creating an immortal hybridoma cell line that can produce the exact same antibody forever (10, 11, 12, 13). Depending on the use case, both pAb and mAb have their pros and cons (14). Although mAbs excel in high batch-to-batch consistency and are easier to sequence, they can only recognize and bind to a single epitope on an antigen. On the other hand, pAbs are more cost effective to produce and can bind to multiple epitopes on an antigen, which enhances binding sensitivity and makes them more resilient to antigen variations. However, the drawbacks of pAbs include batch-to-batch variability and the challenge of characterizing the pAb samples because of their complexity. mAbs provide high specificity and high batch-to-batch consistency, whereas pAbs yield high sensitivity and a faster timeline. Some combine the advantages of both mAb and pAb, devising a mixture of mAbs to ensure both high specificity and high sensitivity, with one example being the coronavirus disease 2019–neutralizing antibody cocktail (15, 16). Especially in cases where high sensitivity is paramount, such as detecting low-abundance proteins or antigens, and resisting gene escape from mutations, pAbs are preferred. Clinical and research applications of pAbs include antivenoms (17) and ELISA immunoassay (18). Successfully sequencing pAbs can resolve the variability problem, as the resulting pAb sequences can be used to manufacture recombinant antibodies with consistent properties.

Currently, pAb sequencing attempts primarily use the combination of mass spectrometry (MS) and gene templates (19, 20, 21, 22). Specifically, *de novo* peptides are matched to a germline database, and select gene templates are used to guide the assembly of peptides into antibody sequences. Antibodies, especially in the complementarity-determining regions, are highly variable, and a germline database derived from previously studied B cells may not contain a template close enough to the antibodies being sequenced. A true “*de novo*” protein sequencing approach would be preferred as it does not rely on an existing database, reducing the bias of discovered sequences over undiscovered ones and enabling new antibody discovery. Le Bihan *et al*. (23) used MS jointly with B-cell sequencing of the pAb sample, which is a more accurate and precise database. Their method shows promise in sequencing dominant antibodies in a polyclonal sample, and the expressed antibodies have comparable binding affinity and neutralizing capacity to the natural antibodies. However, in cases where the original cell line or complementary DNA is unavailable (24, 25, 26), a purely MS-based solution for *de novo* antibody sequencing is necessary. Approaches for mAb sequencing using only MS include PEAKS AB (ALPS assembly algorithm) (27) and TBNOVO (28). Guthals *et al*. (25) proposed PolyExtend, which aligns bottom–up MS/MS spectra with top–down MS/MS spectra to extend a known sequence segment, helping a user to construct a sequence. They demonstrated the sequencing of two dominant mAbs among a background of pAbs, but their results lacked benchmark validation. The lower binding affinity of their sequenced antibodies compared with the natural antibodies indicated potential sequencing errors. In addition, many current solutions (20, 23, 25, 29) may require the user to manually edit or construct the whole sequenced results, posting restrictions on the user’s expertise in antibodies and proteomics.

**Fig. 1:**
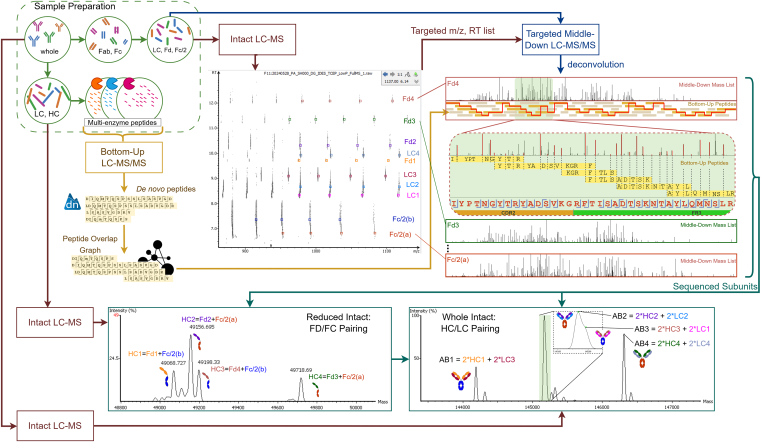
**Overall workflow of pAb *de novo* sequencing with intact mass, targeted middle–down, and bottom–up MS data.** pAb, polyclonal antibody.

Here, we propose PolySeq.AI, an automated and *de novo* pAb sequencing workflow that does not require any gene or protein database. To address the complexity of pAbs, we developed an experimental protocol along with an analytical workflow that integrates intact mass, middle–down, and bottom–up MS data (Fig. 1). Our method offers several key features: peptide *de novo* sequencing from bottom–up data, protein *de novo* sequencing through the integration of middle–down and bottom–up data, and proteoform characterization using intact mass deconvolution. By analyzing multiple levels of MS data, our workflow accumulates sequence information from the peptide to protein level, refining the *de novo* pAb sequences until full coverage and complete accuracy is achieved. The entire process is *de novo* and automated; that is, no database/template or manual intervention is required.

We evaluated PolySeq.AI using a mixture of mAbs generated from the HB-95 cell line as well as a mixture of four well-characterized mAbs. The results showed that our *de novo* antibody sequences achieved 100% bottom–up peptide coverage and 99% to 100% sequencing accuracy for both experiments. These sequences were supported by fragment ions from both bottom–up and middle–down data, and their total masses closely matched the measured intact masses within reasonable tolerance. Furthermore, we used the *de novo* sequences to produce recombinant proteins and validated their functionality through Western blot and immunopeptidome assays. Overall, our PolySeq.AI workflow provides a comprehensive profile of the pAb proteoforms, demonstrating high residue-level sequence accuracy.

## Experimental Procedures

### Method Outline

Figure 1 illustrates the overall workflow of our proposed sequencing method, from sample preparation to data collection, and then to software data processing in PolySeq.AI. During sample preparation (*top-left* in Fig. 1), several differently treated samples were prepared. First, antibodies were deglycosylated, then cleaved at the hinge region using either IdeS (immunoglobulin G–degrading enzyme of *Streptococcus pyogenes*) (30) (at the lower hinge region with the motif “LLG|G”) or SpeB (31) (at the flexible hinge region, with varied motifs depending on the antibody subtype), depending on the experiment, resulting in F(ab)2 and Fc regions, which were further reduced into antibody subunits: light chain (LC), Fd, and Fc/2. For the following sections, we will refer to this sample with LC, Fd, and Fc/2 as the “subunit” sample. The subunit sample was used to collect middle–down data, as the molecules’ masses fall in the range of 20,000 to 30,000 Da, allowing middle–down acquisition to sufficiently fragment the subunits. From deglycosylated antibodies, without IdeS or SpeB cleavage, we reduced the sample to obtain LCs and heavy chains (HCs). For bottom–up data collection, we reduced and alkylated deglycosylated antibodies and then used multiple enzymes (chymotrypsin, elastase, pepsin, and trypsin; Promega Corporation) to produce samples of peptides for bottom–up data collection.

Intact masses were our first insight into the antibody sample, providing an overview of the major mass-detectable antibody forms. Intact LC–MS data were collected for the whole (deglycosylated), reduced, and subunit samples. The different levels of intact data helped us build antibodies, pairing the Fd and Fc/2 subunits into HCs, and pairing LC and HC into antibodies (*bottom* of Fig. 1). In addition, we also used subunit intact mass to help in the targeted middle–down data collection. Following intact deconvolution, we obtained a list of nonoverlapping features (a feature is a window of *m/z* and retention time [RT] for a specific charge of a deconvoluted mass) for the subunit intact data (*top–center* in Fig. 1). From each selected feature, the highest-intensity *m/z* and the corresponding RT are selected, resulting in an inclusion list to guide targeted middle–down precursor selection.

Targeted middle–down experiment was then performed on the subunit sample, using the nonoverlapping inclusion list, with the electron transfer dissociation fragmentation method. The resulting LC–MS/MS data were deconvoluted, and the spectra from the precursors of the same subunit mass were grouped. For each group, the deconvoluted peaks from the spectra in that group were aggregated into one mass list. Hence, from targeted middle–down experiment, we acquired a mass list for each subunit detected from intact mass.

Bottom–up LC–MS/MS data were collected from the differently digested peptide samples. Different enzymes were used to increase peptide overlaps. We used both higher energy collision dissociation and electron transfer/higher energy collision dissociation (EThcD) fragmentation methods. PEAKS *de novo* search (32) was used to generate peptide sequences from the bottom–up data. We built a peptide overlap graph from *de novo* peptides, adding an edge between two peptides if they overlap by at least three amino acids (AAs).

For sequencing, one subunit was sequenced at a time, combining bottom–up and middle–down data. Bottom–up peptides were assembled into paths from the overlap graph, whereas deconvoluted middle–down mass list for the current subunit was used to score paths that best match middle–down fragments (*right side* of Fig. 1). For each subunit’s sequencing step, since we did not know which type the subunit belongs to, we attempt to construct three sequences: an LC, an Fd, and an Fc/2. For each type, we used a different set of starting peptides belonging to the subunit type. When attempting to construct a sequence, we built from left to right, and then from right to left, finally merging the two directions. After that, the SPIDER variant search was then performed on the sequenced subunits to correct isobaric errors, such as swapped AAs. From the three constructed sequences for each subunit, we selected the one with the highest number of middle–down fragment mass matches. Finally, intact mass from different samples was used to group subunits into antibodies.

### Experimental Design and Statistical Rationale

**Fig. 2:**
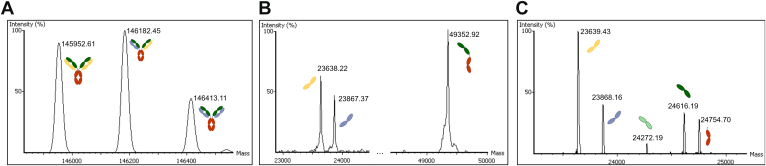
**HB-95 deconvoluted intact mass results.***A*, whole deglycosylated antibodies (*B*) reduced heavy and light chains (LCs) (*C*) LC, Fd, Fc/2 subunits.

Our first experiment as a proof of concept is a purified antibody sample produced by the HB-95 cell line, which specifically targets human leukocyte antigen (HLA)-A, -B, and –C molecules. The commercially available mAb W6/32 is derived from the HB-95 cell line, but for this experiment, we used the mAb sample directly from the cell line rather than the W6/32 mAb. For the following portion of the article, we will refer to this experiment and sample as the “HB-95 sample.” Initially, we attempted to sequence the mAb with the mAb sequencing software PEAKS AB 3.5. Bottom–up peptide assembly generated one HC and one LC, both with full peptide coverage (Supplementary Section S1.1). However, a variant search revealed many high-quality peptides (average local confidence [ALC] above 90%) as variants of the LC (Supplementary Section S1.2). Upon further investigation with intact mass data, we determined that there are three major antibody forms in the antibody sample produced by HB-95. Two LCs and one HC yield three combinations of antibodies, as seen in Figure 2. It has been shown that two LCs and one HC could result from a hybridoma (33), which would be the case for our HB-95 sample. In light of the failure to sequence all the chains using a sequencing software specifically designed for mAbs, we used this experiment as a test for our polyclonal sequencing software.

To evaluate and verify our sequencing results, there are several metrics and approaches. First, as an internal quality control, our sequenced results must have mass values within error tolerance of the observed intact masses. In addition, both bottom–up and middle–down data must support the sequences generated (more detail in the *Results* section). Second, we could use the National Center for Biotechnology Information BLAST tool to search against other published protein sequences. The BLAST results, if matched identically, give us insight into the source, reference, and the validity of our sequences. Finally, we can test the functionality of the expressed antibodies from our sequenced results. Since we know that the HB-95 cell line produces antibodies that capture the HLA-I complexes, we can produce recombinant antibodies from our constructed sequences and examine how they capture HLA-I complexes *via* a Western blot study.

For a more complex experiment, we used a mixture of four commercially known mAbs to simulate a pAb sample. We mixed bevacizumab (BEV, Avastin), adalimumab (ADA, Humira), rituximab (RIT; MabThera), and trastuzumab (TRA; Herceptin) with a ratio of 3:2:1:1. The validation of the four mAb (4mAb) samples is straightforward, as the sequences are known. Sequencing accuracy is calculated as the number of correctly identified AAs divided by the length of the sequenced result. Supplementary Section S2 includes the known sequences of the four antibodies as well as a multiple sequence alignment for the LCs and HCs. Significant sequence variations were observed among the four antibodies, particularly in the complementarity-determining regions of both the HCs and LCs, as well as in the framework region 3 of the HCs. Furthermore, RIT’s framework regions differed significantly from those of the other three antibodies.

To further test the limits of our solution against more extreme mixture ratios, we devised an experiment by mixing the USP1, USP2, and NIST antibodies in different ratios of 3:3:1, 5:5:1, and 10:10:1. An LC–MS intact analysis of the different ratio samples gave us insight into the sensitivity of our approach. Details of this experiment as well as the results are included in Supplementary Section S10.

### Sample Preparation and Data Collection

#### Antibody Mixture Procurement

The HB-95 cell line (Research Resource Identifier: RRID_7872; American Type Culture Collection) was maintained in serum-free medium (Shanghai OPM Biosciences; H081801-001) supplemented with 1× penicillin–streptomycin solution (Gibco) and grown at 37 °C with 5% CO_2_. Cell cultures were passaged at a 1:10 ratio until cell concentrations reached ∼1 × 10 (6). Antibody was purified with protein G beads following the manufacturer’s protocol (GenScript, L00209-100). As a quality control for HB-95 antibody purification, Western blot detection was performed with monomorphic determinants on HLA-A, -B, and -C molecules as described (34).

For the experiment with 4mAbs, we obtained four commercially available mAbs: BEV (Avastin), ADA (Humira), RIT (MabThera), and TRA (Herceptin) and mixed them in a ratio of 3:2:1:1.

#### Sample Preparation

For both HB-95 and 4mAb experiments, we generate the following samples: (1) deglycosylated antibody sample for intact analysis, (2) reduced sample for intact analysis, (3) subunit samples digested with the SpeB (for the HB-95 experiment) or IdeS (for the 4mAb experiments) protease and reduced for both intact and middle–down analysis, and (4) reduced and alkylated sample digested with different enzymes for bottom–up analysis. In this section, we describe the sample preparation conditions for each of the four samples.

##### Deglycosylated Antibody

Twenty micrograms of purified antibody were diluted to a final concentration of 1 μg/μl with a 50 mM final concentration of Tris (pH 8.0) buffer. The N-linked glycans on the antibody constant region were removed by adding 0.2 μl PNGase F (NEB; 15000U, P0705S) at 37 °C, incubated overnight, dried down, and then resuspended in 0.1% formic acid prior to LC–MS analysis.

##### Reduced Sample

After deglycosylation, the antibody was treated with 25 mM Tris(2-carboxyethyl) phosphine hydrochloride (Sigma) and incubated at 37 °C for 30 min, then dried down, and resuspended in 0.1% formic acid (Sigma) prior to LC–MS analysis.

##### Subunit Sample

From the deglycosylated HB-95 sample, the antibody was treated with 20 U of SpeB (Genovis, FabULOUS) for 1 h at 37 °C to cleave the HC hinge region and generate Fab and Fc fragments. For the 4mAb mixture sample, after deglycosylation, the mixture was treated with 20 U of IdeS (Promega) for 30 min at 37 °C to cleave after the HC hinge region to generate F(ab)2 and Fc fragments. For both experiments, after digestion, the sample was reduced with 5 mM Tris(2-carboxyethyl) phosphine hydrochloride, dried down, and then resuspended in 0.1% formic acid (Sigma) prior to LC–MS/MS analysis.

##### Bottom–Up Peptide Sample

Purified HB-95 antibody was reduced with 10 mM DTT, alkylated with 20 mM iodoacetamide, acetone precipitated, and digested with either MS-grade pepsin, elastase, chymotrypsin, or trypsin (Promega Corporation). Digested samples were lyophilized, resuspended in 0.2% TFA, and then desalted using a homemade C18 spin column.

#### Data Collection With MS

As mentioned previously, the whole deglycosylated, reduced, and subunit samples were used to conduct LC–MS intact analysis, the subunit sample was used to conduct LC–MS/MS middle–down analysis, and the digested peptide samples were used to conduct LC–MS/MS bottom–up analysis. Detailed MS instrumentation and methods are included in Supplementary Section S3.

### Sequencing Algorithm

#### Feature-Based Intact Deconvolution for Proteoform Identification

Intact deconvolution is used in the following three ways: (1) to identify mass-detectable proteoform masses, (2) to guide targeted middle–down experiments by providing an inclusion list, and (3) to group antibodies from sequenced subunits. As mentioned in *Method Outline* section, three intact samples were collected: whole deglycosylated, reduced, and subunits.

For each intact sample, the intact deconvolution algorithm can be broken down into three steps: First, a sliding window accumulates neighboring spectra along RT by merging peaks of the same *m/z*. The default window size was set at 0.1 min but can be adjusted according to the experiment and data. Second, we deconvoluted each merged spectrum by assigning charges to each peak and calculating masses from the *m/z* and charge, as outlined in UniDEC (35). Third, we combined the results from all the merged spectra, generating confident proteoform masses that have a continuous stretch along RT. Each reported mass is associated with a list of “features,” which are windows of *m/z* and RT that represent a particular mass at a particular charge. We required the reported masses to have consecutively charged supporting features. More details on the intact deconvolution algorithm are included in Supplementary Section S4.

To aid targeted middle–down experiment, our software generated an inclusion list after subunit intact mass deconvolution: a list of features without *m/z* and RT overlaps for each identified proteoform mass. The inclusion list was generated in a greedy fashion: we sorted the features by intensity from highest to lowest, then picked the features without overlaps with the previously processed features. For each subunit mass, four features were selected in the inclusion list. The inclusion lists for the HB-95 and 4AB subunit samples are included in Supplementary Section S5.

#### Middle–Down Deconvolution and Preprocessing

Traditional untargeted middle–down or top–down experiemnts is conducted with data-dependent acquisition. One observation during our experiments is that low-intensity precursors may not be selected for MS2, or only one spectrum with unsatisfactory fragmentation is produced for a particular precursor. Since our method requires rich middle–down fragmentation, we used targeted middle–down approach, which selects the targeted precursors purposefully, and allows multiple spectra to be generated for each precursor. Merging peaks from spectra belonging to the same precursor increases fragment ion coverage.

From intact deconvolution, we obtained a list of four precursors in the same RT range for each identified mass. Due to instrument limitations, we conducted four LC–MS/MS runs with the same settings and columns but each with a different input list of precursors, yielding four data files. During data processing, each MS2 spectrum was read along with the corresponding precursor information. Having grouped the MS2 spectra by their precursors, we used the TopFD (36) software, particularly the MS-Deconv algorithm (37), to deconvolute middle–down MS2 spectra, obtaining a list of neutral fragment ion masses for each precursor. We then grouped the results of the four precursors (from the four files) corresponding to the same subunit mass into one fragment ion mass list. Since the fragmentation method for middle–down approach was electron transfer dissociation, the fragment ion mass list for each subunit mass included *c* and *z* ions.

For each subunit mass, we generated two top–down mass lists: one assuming all fragment ion masses are *c* ions, which are then converted to neutral prefix residue masses (PRMs); and the other assuming all fragment ion masses are *z*-ions, which are converted to neutral suffix residue masses (SRMs).

#### Bottom–Up Peptide *De Novo* Sequencing and Processing

The raw bottom–up LC–MS/MS data were imported into PEAKS for *de novo* sequencing. The resulting *de novo* peptide-spectrum matches (PSMs) were filtered with an ALC score above 0.7 and continuous unconfident residue length no more than 4. Using the *de novo* peptides, we constructed an overlapping bidirectional graph, preserving post-translational modifications identified during the *de novo* search. To determine the maximum overlap between two peptides, we allowed certain isobaric differences within the overlapped region. We applied the concept of a “mass block” (38) to represent equal-mass short segments (with a maximum length of 2) in both peptides. For example, when comparing the peptides SEQVENCE and EGGECPEP below, the alignment reveals the overlapping region as [E][N/GG][CE/EC], consisting of three mass blocks, each no longer than two AAs.

SEQV E N CE

E GG EC PEP

The overlap graph was constructed with *de novo* peptides as vertices, where an edge was drawn between two peptides if they shared an overlap of at least three mass blocks. Using this graph, we further refined each peptide’s local confidence scores as follows:

● For a peptide P, we considered all peptides Qᵢ that could extend P to the right. The local confidence scores of Qᵢ in the overlapping regions with P were accumulated into P’s new local scores.
● In cases where the overlap formed a two-residue mass block, the ALC score was used.
● Similarly, for peptide Rⱼ that could extend P to the left, their overlapping scores were also accumulated into P.

This process ensured that the updated local confidence scores at each residue reflected the entire bottom–up spectral coverage, rather than being limited to a single spectrum. Traditional bottom–up PEAKS *de novo* sequencing calculates residue confidence based on individual PSMs. When a spectrum exhibits poor fragmentation, particularly near the N or C termini, the local confidence for residues in those regions is low. By aggregating data from overlapping peptides, the positional confidence for each AA is improved. If another overlapping PSM provides strong fragmentation coverage of the same residues, the combined data boost the overall local confidence.

An additional step that we conducted during bottom–up processing was determining the set of start and end peptides for each subunit type: LC, Fd, and Fc/2. In order for the assembly of peptides in the sequencing step, we needed a small subset of peptides that can be sequence starts, and respectively sequence ends, for the reverse sequencing direction. The task then was to determine which group a *de novo* peptide belongs to: LC-START, LC-END, Fd-START, Fd-END, Fc/2-START, Fc/2-END, or OTHER. Regarding this issue, several studies (26, 39, 40) suggest that antibody LC and HCs’ beginnings and endings are relatively conserved and follow general patterns. Therefore, we trained a transformer-classifier model to do this task, the general idea of which came from the highly successful protein deep learning model, evolutionary scale modeling (41). We acquired immunoglobulin antibody sequences from the IMGT database (42), totaling 1058 HC and 3218 LC sequences. This antibody sequence database was not used in any other part of the sequencing program. We randomly split the sequences into train, validation, and testing sets with a ratio of 7:1:2. For each protein sequence, we randomly sampled starting, middle, and ending peptides for supervised learning. Evaluation with the testing dataset results in 99.7% classification accuracy, calculated as the number of correctly labeled peptides divided by the total peptides in the testing set. More details regarding database collection, model structure, training, and testing conditions are included in Supplementary Section S6.

#### *De Novo* Antibody Sequencing With Bottom–Up and Middle–Down Processing

**Fig. 3:**
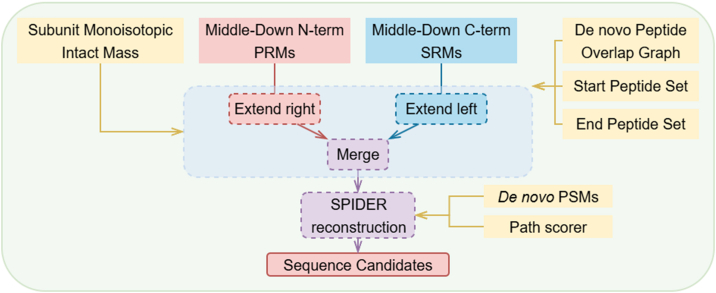
**Sequencing workflow for one subunit, attempt of one subunit type**.

For each subunit, we had a monoisotopic mass, a list of middle–down fragment ion masses, a bottom–up peptide overlap graph, and sets of start and end peptides. At this stage, the specific type of subunit (LC, Fd, or Fc/2) was unknown. Therefore, we sequenced the subunit three times, each time using one of the three sets of start and end peptides. From the resulting sequences, we selected and reported the one with the highest number of middle–down matches. The following section describes the process of sequencing for one subunit type, as outlined in Figure 3. Supplementary Section S7 includes an example of sequencing one subunit (from the 4mAb mixture experiment, the sequencing of ADA Lc), including sequence candidates of each direction, merged results, and the final best candidate.

During middle–down processing, we generated two lists: PRMs (N-terminal fragment masses) and SRMs (C-terminal fragment masses). One could convert all the SRMs to PRMs by subtracting from the total precursor mass, but inaccuracies found in the intact mass introduce errors in the converted masses. For example, a sequence’s total mass is 23,000 Da, and the last nine AAs’ total mass is 1000 Da. An observed SRM is 1000.05 Da. The deconvoluted intact mass is 23,001 Da. Were we to convert the SRM into PRM, the mass error would increase from 0.05 Da (50 ppm) to 0.95 Da (950 ppm). To avoid introducing more noise into sequencing, we opt to use the PRM list to sequence from left (N terminal) to right (C terminal) and the SRM list to sequence in the reverse direction. We found that most deconvoluted middle–down fragment masses ranged from 1000 to 15,000 Da. This indicates that *c*-ions primarily cover the N-terminal half of the subunit, whereas *z*-ions mainly cover the C-terminal half. By using N-terminal PRMs to guide sequencing from the N terminus and extending from left to right, high sequencing accuracy can be ensured for the left half of the subunit, and similarly, C-terminal SRMs can ensure accuracy for the right half. Merging forward and reverse sequencing extensions will therefore maximize middle–down coverage across the entire sequence.

Using a peptide overlap graph with local confidence scores, a middle–down PRM list, and a monoisotopic mass, we applied a dynamic programming (DP) algorithm to extend the sequence forward. We constructed a DP table, where each column represents a discretized mass index. Our default discretization width was set to 0.1 Da, meaning that a mass of 111.2 Da would have an index of 1112. At each mass index, we stored a bounded priority queue of extended paths whose total mass matches that index. Each path is represented as a linked list formed from overlapping peptides. To manage computational complexity, we limited the priority queue to a maximum of 10 entries. For a path p with total mass m, we placed it in the priority queue at the corresponding discretized index I(m) and assigned it a score of $s\left(p\right)=T+\left(N_{A}-N_{U}\right)$, where T is the number of matching middle–down fragments. $N_{A}$ denotes the average number of residues given the path mass, calculated by the current mass divided by the averagine residue mass. $N_{U}$ is the number of unconfident residues. Confidence scores for each residue were calculated during overlap graph construction. Therefore, the path score takes into account both middle–down matches and confident positions.

The set of peptides that could serve as subunit start points was first populated into the DP table. Then, at each mass index and for each path in the priority queue, we used the forward graph to identify candidate peptides that could be extended. For each candidate, we computed the new path mass, the number of matching middle–down fragments, and the number of unconfident residues before adding the new extended path to the DP table. Once the DP table was fully populated, we identified paths whose total mass was close to the intact subunit mass, allowing for some error tolerance (4 Da). We then performed backtracking on these paths. If a path fell within the desired mass range but ended with a peptide not in the predefined set of ending peptides, a penalty was applied to its score. Finally, the sequences were ranked based on their final path scores, and the top 10 paths were returned. Supplementary Section S7.1 demonstrates one step in building the DP table, including path scores.

The reverse sequencing process follows a similar approach but uses C-terminal middle–down SRMs. In this case, the DP table was initialized with the set of subunit-ending peptides, and sequencing proceeded backward until reaching the set of subunit-start peptides. To merge sequences from both directions, we required that the total mass match the intact subunit mass and that at least one peptide overlap at the joining site. After pairing forward and reverse sequences, we ranked them based on total middle–down matches and reported the top 10 sequences. Examples are shown in Supplementary Section S7.2 and S7.3.

#### Postprocessing With SPIDER Calibration and Ile–Leu Resolution

The next step involved using SPIDER (43) to correct isobaric errors that may have occurred during sequence extension, such as N *versus* GG, SL *versus* TV, or swapped AAs. SPIDER identifies the “true” sequence by comparing *de novo* peptides with a homologous sequence—in this case, our sequenced result—to correct *de novo* errors and detect mutations. To refine the sequence, we used a path scorer based on high-quality *de novo* PSM spectra and focused on segments containing at least two residues with low local confidence scores. We then performed a SPIDER homology search with *de novo* PSMs to identify possible mutations within these segments. To ensure accuracy, we took a consensus of candidate mutations with identical masses. For each potential replacement, we applied the path scorer again to determine whether the substitution improved local confidence. After SPIDER-based reconstruction, we retained the top five candidate sequences ranked by local confidence scores. Supplementary Section S7.4 continues the sequencing example of ADA LC to demonstrate the effect of SPIDER calibration.

The AAs Ile and Leu have the same residue mass and are therefore difficult to distinguish during sequencing. However, many studies (44, 45, 46) demonstrate that EthcD produces *w* and *w*’ ions, as a result of moiety loss from *z* and *z*’ ions. The mass difference between *w* and *z* ions, and *w*’ to *z*’ ions, is distinct for Ile and Leu. Therefore, EthcD spectra can help determine whether a residue is Ile or Leu. During sequencing, we convert every Ile into Leu to simplify the sequencing process. After sequencing, we apply an additional step to examine every position of Leu, to determine which of Ile and Leu it should be. We take the *de novo* PSMs from EthcD fractions that are mapped to this position and take a consensus of the *w* or *w*’ ion evidence for either Ile or Leu. If more Ile evidence is found, we set the residue to Ile and *vice versa*. Supplementary Section S7.5 extends our previous sequencing example to illustrate the Ile and Leu distinction.

#### Antibody Pairing Using Intact Mass Results

From the sequencing step outputs, each subunit mass is linked to one reported sequence belonging to one of the LC, Fd, or Fc/2 subunit types. The remaining step was to group these sequences to reconstruct antibodies consistent with intact MS data.

The subunit mass of an LC is the same as that in the reduced sample. The sum of Fd and Fc/2 masses minus water is equal to the reduced HC mass. We constructed HC sequences by pairing sequenced Fd and Fc/2 subunits, provided their connection was supported by the enzyme digestion rules and bottom–up peptides covered the joint sites. If the mass of a combined HC sequence is equal (within an error tolerance of 4 Da) to an observed reduced HC mass, we save the HC pairing. Once LC and HC sequences were identified, we generated possible antibodies from pairings of LC and HC and matched the expected mass with the observed whole intact mass with error tolerance of 100 ppm. The reason for such a high error tolerance is that we observed disulfide bonds in the reduced sample (meaning that the sample was reduced, but some inner-chain disulfide bonds were reformed) (47). Examples of observed disulfide bonds in the reduced sample are further discussed in the *Results* section for both experiments and further evidenced in Supplementary Tables S8.2 and S9.2.

## Results

### Evaluation of the HB-95 Experiment

Figure 2 shows the deconvoluted intact masses for the three intact samples: whole, reduced, and subunit. Intact mass analysis identified four primary subunit masses, leading PolySeq.AI to construct two LCs, one Fd, and one Fc/2 (Fig. 2*C*). Reduced intact mass results confirm the assembly of Fd and Fc/2 into a complete HC (Fig. 2*B*). Whole intact mass analysis identified three distinct antibody forms, each consisting of different pairings of LC and HC (Fig. 2*A*). One of these antibody forms was found to be bispecific, containing two different LCs and a duplicated HC. In addition, Figure 2*C* shows the presence of a minor subunit antibody form with average mass around 24,272 Da (*light green*), though no middle–down spectra were generated for this variant. During result validation, we observed that the deconvoluted subunit mass matched the expected mass of the Fd region when the SpeB protease cleaved the HC at a minor site, located five AAs upstream of the major cleavage site (31). Detailed intact mass lists with relative abundance are listed in Supplementary Section S8.1.

**Fig. 4:**
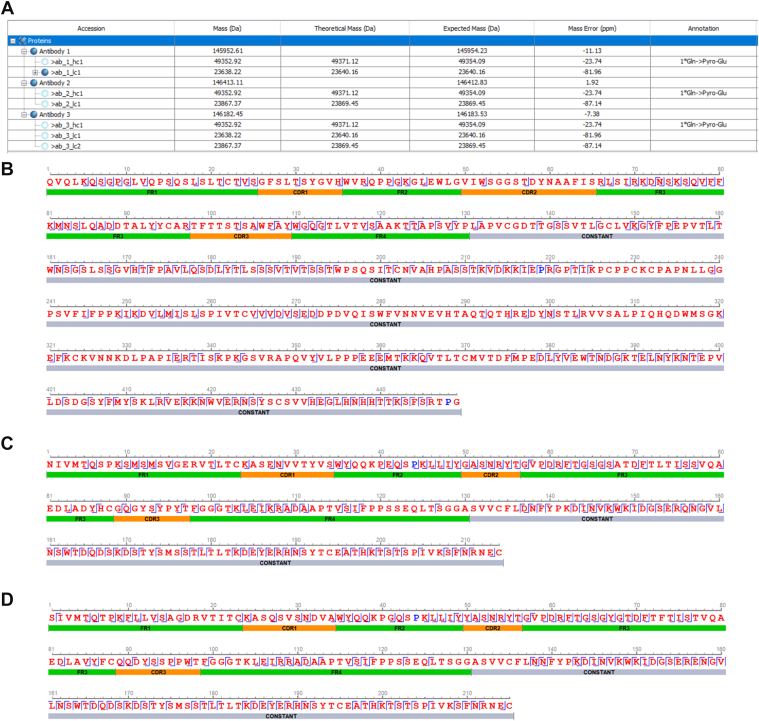
**HB-95 grouping and sequencing results.***A*, antibody groups table with expected and observed mass and annotations. All reported HCs have lysine truncation (not in the table) and pyroglutamic acid from Gln (shown in the table). *B*–*D*, coverage pane for sequenced HC, LC1, and LC2, respectively. Bottom–up fragment ion confidence is indicated by the color of each residue: Red >95%, blue >85%, and black ≤85%. Middle–down fragment ions are labeled between residues as *thin blue bars*. HC, heavy chain; LC, light chain.

From the mass lists, three antibody groups (as seen in Fig. 2) are generated. The other intact mass results from the three samples did not match to form any antibody pairings. Figure 4 displays the grouping and sequencing results. An additional FASTA of the generated sequences is included in Supplementary Section S8.2. From Figure 4, *B*–*D*, we observed complete bottom–up coverage with high fragment ion confidence (above 95% for most residues and above 85% for all residues) for all three sequences. As for middle–down coverage, we can observe long consecutive regions with continuous middle–down fragment ion match. Having defined middle–down coverage to be the number of matched fragment ions divided by the total number of possible (*c*, *z*) fragments with 50 ppm error tolerance (determined based on instrument resolution), we observe the middle–down coverage to be 55.84%, 41.28%, 61.68%, and 64.65% for Fd, Fc/2, LC1, and LC2, respectively. In contrast, the erroneously stitched LC reported by the monoclonal AB sequencing algorithm, as described in the *Experimental Design and Statistical Rationale* section, achieves a middle–down coverage of less than 6% when matched against the middle–down fragments produced by either LC1 or LC2 subunit precursors. Therefore, the bottom–up and middle–down coverage, as well as the mass difference between expected and observed intact masses, indicate that our constructed sequences are highly supported by MS data.

We conducted a BLAST search against the National Center for Biotechnology Information protein database to identify similar sequences reported in previous studies. Our results showed that both of our LCs were a perfect 100% match to two antibody LCs reported in the Protein Data Bank under identification numbers 5H2B_B and 7T0L_K. Our HC variable region matched identically to 7T0L_G, and the HC constant region matched identically to many entries, one of them being 4WEB_H. Interestingly, LC2 and HC variable regions matched to the sequences (7T0L_K and 7T0L_G) from the same source, with the description “HLA-B∗27:05 in complex with the pan-HLA-Ia mAb W6/32”. W6/32 is the commercial mAb that is produced from the HB-95 cell line. The entry for W6/32 did not contain the HC constant region, but from the numerous identical matches in the database for the HC constant region, and also because of the fact that constant regions are highly conserved, we are confident in the entire HC result.

Our sequenced HC and two LCs were used to generate recombinant proteins for further validation. The resulting antibodies were applied to immunoprecipitate HLA-I complexes. The Western blot analysis confirmed the enrichment of HLA-I proteins, as evidenced by distinct and intense bands corresponding to HLA-I HCs (Supplementary Fig. S8.1*A*). Subsequent LC–MS/MS analysis of the eluted immunopeptides attached to HLA-I complexes revealed a characteristic length distribution typical of HLA-I peptides (Supplementary Fig. S8.1*B*). Together, the Western blot and immunopeptidome analyses confirmed the accuracy and functionality of the *de novo* antibodies identified from the HB-95 sample. More information on the validation process with Western blot and immunopeptidome analysis can be found in Supplementary Section S8.3.

### Evaluation of the 4mAb Mixture

There are a total of 10 expected subunits, as bevacizumab (BEV) and TRA share the same Fc region, as do ADA and RIT. Supplementary Section S9.1 includes deconvoluted intact mass lists compared with theoretical masses. Briefly, all the expected masses for all samples were reported, along with some other masses. For example, in the subunit sample, the monoisotopic mass of 23,793.9 Da was reported with 12% relative abundance, which we annotate to be the Fc/2 mass 23,775.9 Da plus 18 (water). All the HCs and Fc/2 subunits were annotated with lysine truncation, and both LC and HC (therefore Fd) of RIT were annotated with pyroglutamic acid from Gln at the protein N terminus.

Curiously, the abundance of the deconvoluted intact masses for each of the whole, reduced, and subunit samples was not in line with the input sample ratio. Particularly, for example, although RIT and TRA have the same ratio in the mixture, the relative abundance of the Fd subunits of the two antibodies is 28.14% and 5.25%, respectively. The abundance calculation is consistent with the LC–MS data (Supplementary Fig. S4.1, *E* and *F*, showing Fd2 from TRA and Fd1 from RIT). This deviation from expected ratios points us to the observation that different molecules exhibit different ionization efficiencies, and sample abundance is not indicative of MS-detectable abundance (48). Though our experiment input is 3:2:1:1, our software demonstrated the sequencing of a subunit with a relative abundance as low as 5%.

A total of five antibody pairings were reported (Table 1), including the four expected pairings and one false identification. One error comes from pairing the ADA HC (49,198.33) with BEV LC (23,449.77), which is within error tolerance (100 ppm) of the whole intact mass of 145,269.05 Da with 10% relative abundance. As previously discussed in “Antibody Pairing Using Intact Mass Results” as well as Supplementary Section S4 regarding the intact deconvolution algorithm, our intact mass error tolerance for antibody pairing is very lenient, because intact mass inaccuracies may stem from multiple factors, such as instrument resolution, isotopic distribution, reformation of disulfide bonds (47), etc.

**Table 1:** 4AB mixture pairing results with deconvoluted average mass in dalton

AB	Chain	Subunit	Subunit mass	Reduced mass	Whole mass
AB1 (TRA)	HC	Fd	25,383.27	49,156.70 (1.6 ppm)	145,166.20 (−5.6 ppm)
		Fc/2	23,791.52		
	LC		23,443.75	23,442.11 (69.9 ppm)	
AB2 (BEV)	HC	Fd	25,945.31	49,718.69 (2.4 ppm)	146,309.38 (−32.1 ppm)
		Fc/2	23,791.52		
	LC		23,451.05	23,449.77 (54.6 ppm)	
AB3 (ADA)	HC	Fd	25,458.32	49,198.33 (32.7 ppm)	145,190.53 (−29.2 ppm)
		Fc/2	23,759.63		
	LC		23,411.85	23,410.93 (39.3 ppm)	
AB4 (RIT)	HC	Fd	25,328.32	49,068.73 (24.7 ppm)	144,187.91 (−39.4 ppm)
		Fc/2	23,759.63		
	LC		23,039.26	23,038.50 (32.1 ppm)	
AB5 (mispaired)	HC	Fd	25,458.32	49,198.33 (32.7 ppm)	145,269.05 (−34.8 ppm)
		Fc/2	23,759.63		
	LC		23,451.05	23,449.77 (54.6 ppm)	

The “subunit mass” column indicates deconvoluted intact mass from the subunit sample. The “reduced mass” and “whole mass” columns indicate deconvoluted intact mass from the reduced and whole samples, respectively, and the values in the parentheses are the mass difference (in ppm) between the calculated masses from the pairing and the observed mass in the column. For HC, the calculated mass is the sum of Fd and Fc/2 minus the average mass of water (18.011 Da). For whole antibodies, the calculated mass is twice the sum of HC and LC minus 16 pairs of disulfide bonds. Five antibodies were reported, with one being mispaired.

Table 2 summarizes each LC’s and HC’s sequencing accuracy along with middle–down coverage. Our *de novo* sequencing results achieved 99% to 100% accuracy and 51% to 61% middle–down coverage. Accuracy is calculated as the number of correctly sequenced residues divided by the total number of expected residues, whereas middle–down coverage calculation is previously described in the HB-95 evaluation section. Supplementary Section S9.2 includes the constructed sequences and their alignments with the ground truth sequences. In short, the discrepancies between the *de novo* and ground-truth sequences were primarily because of isobaric differences, such as N/GG, SL/TV, and I/L. Notably, one I/L assignment differs from the ground truth, found in the BEV LC at position 29, with a reported low confidence. We found one PSM with *w* ion to support L, whereas no PSM with *w* ion to support I. The corresponding PSM with the *w* ion is included in Supplementary Section S9.3.

**Table 2:** Sequencing accuracy (acc.) for LC and HC for the four expected mAbs in mixture and middle–down coverage (MD cov.) for LC, Fd, and Fc/2 subunits for the four mAbs

Sequence	BEV			TRA			ADA			RIT		
	LC	HC		LC	HC		LC	HC		LC	HC	
		Fd	Fc/2		Fd	Fc/2		Fd	Fc/2		Fd	Fc/2
Acc. (%)	99.5	99.6		100	99.5		100	100		99.6	100	
MD cov. (%)	51.9	46.3	54.8	58.6	52.7	54.8	60.3	59.2	57.6	64.8	48.5	57.6

Additional examples of MS spectra, total ion chromatogram, and annotations for the 4mAb experiment can be found in Supplementary Section S11.

## Discussion

*De novo* sequencing of pAb samples is an important task in proteomics and immunology. Although bottom–up MS is widely used for mAb *de novo* sequencing, its application to pAb samples has been limited because of their high complexity. Previous studies have proposed using additional information, such as intact mass, germline databases, antibody knowledgebases, or RNA-sequencing data to assist pAb sequencing (19, 20, 22, 23). In this study, we presented PolySeq.AI, an MS-based workflow that integrates intact mass, middle–down, and *de novo* bottom–up approach for pAb sequencing. By leveraging high-resolution middle–down mass spectra with a targeted approach, our method effectively assembles bottom–up *de novo* peptides while using intact mass data to group and profile antibodies.

We evaluated our proposed method through two experiments: one using an mAb mixture from the HB-95 cell line and another with a mixture of four known mAbs. Our results demonstrated over 99% residue accuracy across all LCs and HCs with complete bottom–up coverage with high local confidence and strong middle–down fragment support. Our automated software enables scientists from all fields to analyze their pAb proteomics data and improves the throughput of pAb analysis, as little to no manual work is required. Furthermore, our *de novo* sequencing algorithm can be adapted to analyze even more complex polyclonal or protein mixture samples.

While our method addressed one complex antibody mixture case, some other improvements could significantly expand PolySeq.AI’s capabilities and broaden its applications. One main challenge lies within the limitations of MS. Our method heavily relies on intact mass to direct targeted middle–down experiments and antibody pairing. For a pAb or mixture sample where a target antibody’s intact mass signal is too low, our solution would not be able to identify and consequently sequence the antibody (as seen in Supplementary Section S10). This may be addressed with affinity purification (14), RT separation, and fractionation, such as GELFrEE (49, 50), or the advancement of MS instruments. Strong middle–down fragmentation signals are also needed to achieve good sequencing results. The way we obtained good fragmentation in PolySeq.AI is *via* targeted middle–down experiment. Several MS technological advances that improve middle–down coverage may be investigated and applied, such as proton transfer charge reduction (51), ultraviolet photodissociation fragmentation (52), and so on.

Another direction for future improvement is within our proposed algorithm. For example, intact mass analysis for Fab regions may be used to pair Lc and Fd as an added layer of confirmation. Our current method utilizes deep learning to classify peptides during bottom–up peptide processing. We can extend the use of deep learning models to help in the assembly and sequencing of antibody subunits, either in sequence prediction or candidate scoring.

pAb’s advantages are clear: they are cheaper to produce, can bind to multiple epitopes on an antigen, and are sensitive to low-abundance antigens. On the other hand, the drawbacks of pAbs include the possibility of binding to unwanted epitopes as well as batch-to-batch variability resulting from finite supply. Affinity purification can be conducted to reduce the risk of unwanted bindings and recombinant renewable pAbs (8). Following the accurate sequencing of pAbs is a promising approach to supply pAbs endlessly. Being able to sequence antibody mixtures, our protocol shows promise in the development of recombinant pAbs, which can be applied to replace or enhance traditional pAbs in various diagnostic and therapeutic applications, such as ELISA (18), antivenom (17), rabies vaccines (53), etc.

## Data Availability

The datasets used in this article have been deposited to the ProteomeXchange Consortium *via* the PRIDE partner repository with the dataset identifier PXD063526, token OJ66W3eAfiR1. The data will be made public when the article is published.

## Supplemental Data

This article contains supplemental data.

## Conflict of Interest

L. X., W. L., S. Z., N. H. T., Z. C., A. A., K. H., X. Z., W. S., L. L., Z. W., and B. S. are employees of Bioinformatics Solutions, Inc, Waterloo, Ontario, Canada. J. M. and C. P. are employees of BaizhenBio, Inc, Wuhan, China. The authors declare no competing interests.
