# Supplementary material for: Sequencing of Polyclonal Antibodies by Integrating Intact Mass, Middle–Down, and De Novo Bottom–Up Mass Spectrometry
Source: Mol Cell Proteomics. 2025 Oct 13;24(11):101088. doi: 10.1016/j.mcpro.2025.101088 (PMC12639833; doi:10.1016/j.mcpro.2025.101088)
Supplement: Supplementary Materials [file mmc1.pdf]

S1 HB-95 Sample Sequencing Results from PEAKS AB 3.5 (Monoclonal Antibody Sequencing)

S1.1 Constructed Sequences Coverage

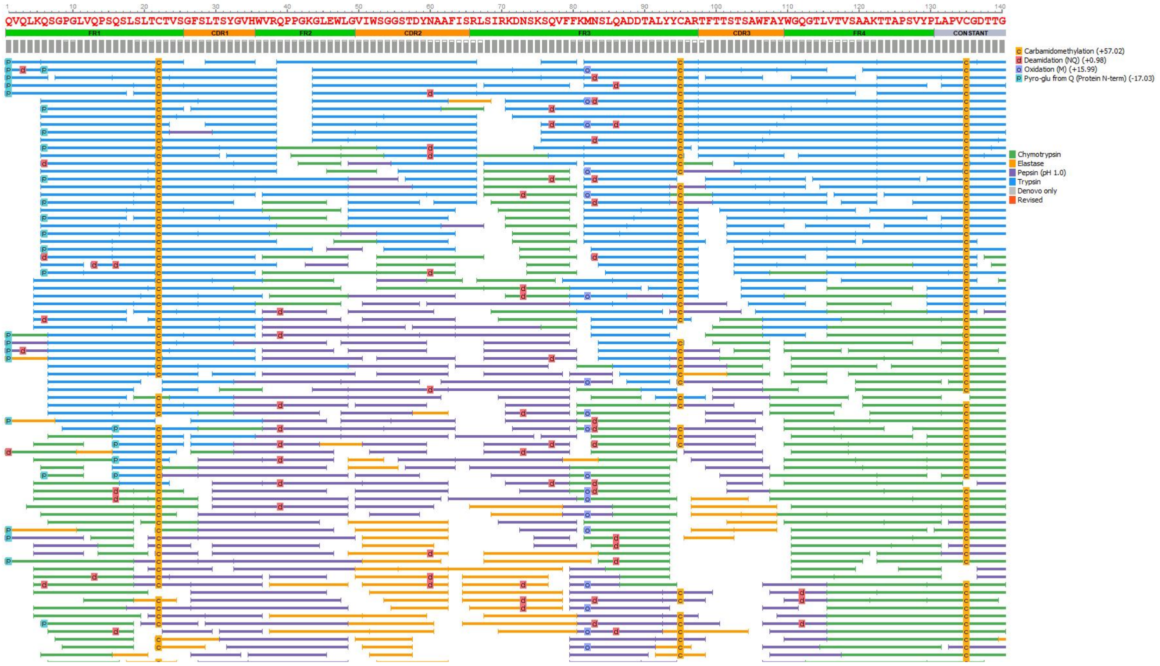

Figure S1.1: Bottom-up coverage for sequenced heavy chain variable region. Sequence was constructed by PEAKS AB 3.5 monoclonal antibody sequencing software, using all bottom-up de novo and DB search peptides from the HB-95 bottom-up samples (with enzymes Chymotrypsin, Elastase, Pepsin and Trypsin). This figure shows that full bottom-up coverage of sequenced heavy chain variable region.

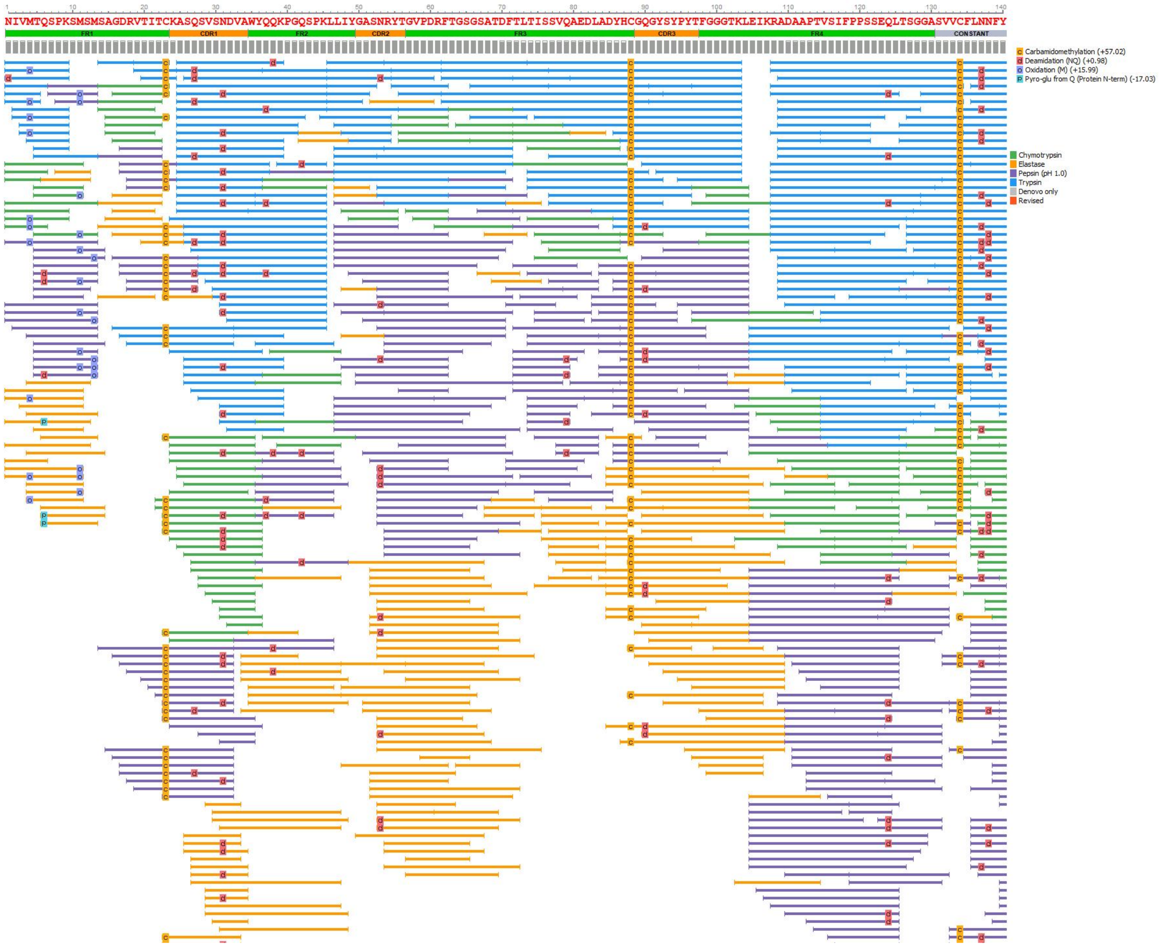

Figure S1.2: Bottom-up coverage for sequenced light chain variable region. Sequence constructed under the same condition as Figure S1.1. This figure shows that the entire sequenced light chain variable region has good bottom-up coverage, even though from later findings we know that the sequence is in fact incorrect.

S1.2 Light Chain Variance Pane

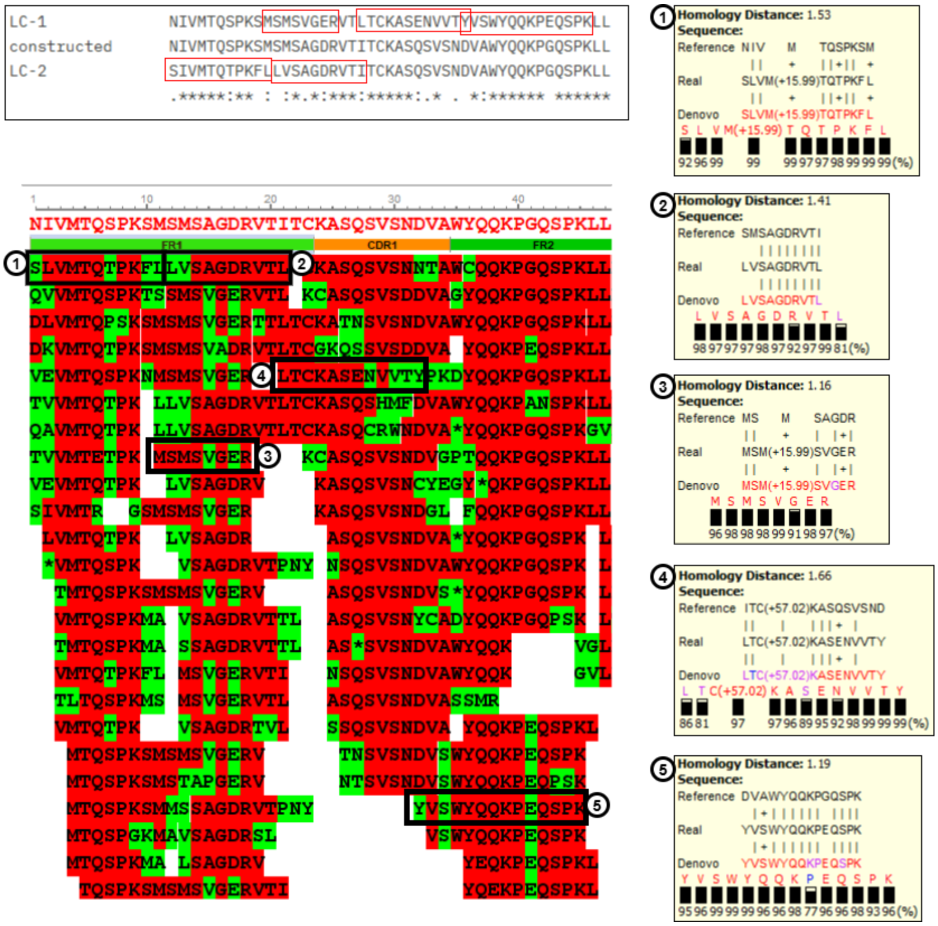

Figure S1.3: Top-Left: Sequence alignment of the true sequences LC1, LC2, and the constructed sequence. Alignment shows that the constructed sequence stitched the true sequences at the common amino acid S on position 14. Bottom-Left: Variants pane for sequenced light chain fragment. Sequence was constructed by the same condition as Figure S1.1. Boxed regions with numbers indicate specific peptides to examine further. Right: Detailed SPIDER variants search results for selected de novo peptides. Peptides numbered 1 and 2 support LC-2 as a variant to the constructed region in the range 1 to 21; peptides 3 to 5 support LC1 as a variant in the range 11 to 45. However, without knowing the true sequences LC1 and LC2, it is difficult to derive the correct sequence(s) from the variants.

S2 Four mAb Mixture True Sequences With Multiple Alignment

```
>Bevacizumab Light chain
DIQMTQSPSSLSASVGDRVTITCSASQDISNYLNWYQQKPGKAPKVLIYFTSSLHSGVPSRFSGSGSGTDFTLTISSLQPEDFATYYCQQYSTV
PWTFGQGTKVEIKRTVAAPSVFIFPPSDEQLKSGTASVVCLLNNFYPREAKVQWKVDNALQSGNSQESVTEQDSKDSTYSLSSTLTLSKADYEK
HKVYACEVTHQGLSSPVTKSFNRGEC
>Adalimumab Light chain
DIQMTQSPSSLSASVGDRVTITCRASQGIRNYLAWYQQKPGKAPKLLIYAASTLQSGVPSRFSGSGSGTDFTLTISSLQPEDVATYYCQRYNRA
PYTFGQGTKVEIKRTVAAPSVFIFPPSDEQLKSGTASVVCLLNNFYPREAKVQWKVDNALQSGNSQESVTEQDSKDSTYSLSSTLTLSKADYEK
HKVYACEVTHQGLSSPVTKSFNRGEC
>Rituximab Light chain
QIVLSQSPAILSASPGEKVTMTCRASSSVSYIHWFQQKPGSSPKPWIYATSNLASGVPVRFSGSGSGTSYSLTISRVEAEDAATYYCQQWTSNP
PTFGGGTKLEIKRTVAAPSVFIFPPSDEQLKSGTASVVCLLNNFYPREAKVQWKVDNALQSGNSQESVTEQDSKDSTYSLSSTLTLSKADYEKH
KVYACEVTHQGLSSPVTKSFNRGEC
>Trastuzumab Light chain
DIQMTQSPSSLSASVGDRVTITCRASQDVNTAVAWYQQKPGKAPKLLIYSASFLYSGVPSRFSGSRSGTDFTLTISSLQPEDFATYYCQQHYTT
PPTFGQGTKVEIKRTVAAPSVFIFPPSDEQLKSGTASVVCLLNNFYPREAKVQWKVDNALQSGNSQESVTEQDSKDSTYSLSSTLTLSKADYEK
HKVYACEVTHQGLSSPVTKSFNRGEC
>Bevacizumab Heavy chain
EVQLVESGGGLVQPGGSLRLSCAASGYTFTNYGMNWVRQAPGKGLEWVGWINTYTGEPTYAADFKRRFTFSLDTSKSTAYLQMNSLRAEDTAVY
YCAKYPHYYGSSHWYFDVWGQGTLVTVSSASTKGPSVFPLAPSSKSTSGGTAALGCLVKDYFPEPVTVSWNSGALTSGVHTFPAVLQSSGLYSL
SSVVTVPSSSLGTQTYICNVNHKPSNTKVDKKVEPKSCDKTHTCPPCPAPELLGGPSVFLFPPKPKDTLMISRTPEVTCVVVDVSHEDPEVKFN
WYVDGVEVHNAKTKPREEQYNSTYRVVSVLTVLHQDWLNGKEYKCKVSNKALPAPIEKTISKAKGQPREPQVYTLPPSREEMTKNQVSLTCLVK
GFYPSDIAVEWESNGQPENNYKTTPPVLDSDGSFFLYSKLTVDKSRWQQGNVFSCSVMHEALHNHYTQKSLSLSPGK
>Adalimumab Heavy chain
EVQLVESGGGLVQPGRSLRLSCAASGFTFDDYAMHWVRQAPGKGLEWVSAITWNSGHIDYADSVEGRFTISRDNAKNSLYLQMNSLRAEDTAVY
YCAKVSYLSTASSLDYWGQGTLVTVSSASTKGPSVFPLAPSSKSTSGGTAALGCLVKDYFPEPVTVSWNSGALTSGVHTFPAVLQSSGLYSLSS
VVTVPSSSLGTQTYICNVNHKPSNTKVDKKVEPKSCDKTHTCPPCPAPELLGGPSVFLFPPKPKDTLMISRTPEVTCVVVDVSHEDPEVKFNWY
VDGVEVHNAKTKPREEQYNSTYRVVSVLTVLHQDWLNGKEYKCKVSNKALPAPIEKTISKAKGQPREPQVYTLPPSRDELTKNQVSLTCLVKGF
YPSDIAVEWESNGQPENNYKTTPPVLDSDGSFFLYSKLTVDKSRWQQGNVFSCSVMHEALHNHYTQKSLSLSPGK
>Rituximab Heavy chain
QVQLQQPGAELVKPGASVKMSCKASGYTFTSYNMHWVKQTPGRGLEWIGAIYPGNGDTSYNQKFKGKATLTADKSSSTAYMQLSSLTSEDSAVY
YCARSTYYGGDWYFNVWGAGTTVTVSAASTKGPSVFPLAPSSKSTSGGTAALGCLVKDYFPEPVTVSWNSGALTSGVHTFPAVLQSSGLYSLSS
VVTVPSSSLGTQTYICNVNHKPSNTKVDKKAEPKSCDKTHTCPPCPAPELLGGPSVFLFPPKPKDTLMISRTPEVTCVVVDVSHEDPEVKFNWY
VDGVEVHNAKTKPREEQYNSTYRVVSVLTVLHQDWLNGKEYKCKVSNKALPAPIEKTISKAKGQPREPQVYTLPPSRDELTKNQVSLTCLVKGF
YPSDIAVEWESNGQPENNYKTTPPVLDSDGSFFLYSKLTVDKSRWQQGNVFSCSVMHEALHNHYTQKSLSLSPGK
>Trastuzumab Heavy chain
EVQLVESGGGLVQPGGSLRLSCAASGFNIKDTYIHWVRQAPGKGLEWVARIYPTNGYTRYADSVKGRFTISADTSKNTAYLQMNSLRAEDTAVY
YCSRWGGDGFYAMDYWGQGTLVTVSSASTKGPSVFPLAPSSKSTSGGTAALGCLVKDYFPEPVTVSWNSGALTSGVHTFPAVLQSSGLYSLSSV
VTVPSSSLGTQTYICNVNHKPSNTKVDKKVEPKSCDKTHTCPPCPAPELLGGPSVFLFPPKPKDTLMISRTPEVTCVVVDVSHEDPEVKFNWYV
DGVEVHNAKTKPREEQYNSTYRVVSVLTVLHQDWLNGKEYKCKVSNKALPAPIEKTISKAKGQPREPQVYTLPPSREEMTKNQVSLTCLVKGFY
PSDIAVEWESNGQPENNYKTTPPVLDSDGSFFLYSKLTVDKSRWQQGNVFSCSVMHEALHNHYTQKSLSLSPGK
```

```
Bevacizumab_LC/1-21    1 DIQMTQSPSSLSASVGDRVTITCSASQDISNYLNWYQQKPGKAPKVLIYFTSSLHSGVPSRFSGSGSGTDFTLTISSLQP  80
Adalimumab_LC/1-214    1 DIQMTQSPSSLSASVGDRVTITCRASQGIRNYLAWYQQKPGKAPKLLIYAASTLQSGVPSRFSGSGSGTDFTLTISSLQP  80
Rituximab_LC/1-213     1 QIVLSQSPAILSASPGEKVTMTCRASSSV-SYIHWFQQKPGSSPKPWIYATSNLASGVPVRFSGSGSGTSYSLTISRVEA  79
Trastuzumab_LC/1-21    1 DIQMTQSPSSLSASVGDRVTITCRASQDVNTAVAWYQQKPGKAPKLLIYSASFLYSGVPSRFSGSRSGTDFTLTISSLQP  80

Bevacizumab_LC/1-21   81 EDFATYYCQQYSTVPWTFGQGTKVEIKRTVAAPSVFIFPPSDEQLKSGTASVVCLLNNFYPREAKVQWKVDNALQSGNSQ 160
Adalimumab_LC/1-214   81 EDVATYYCQRYNRAPYTFGQGTKVEIKRTVAAPSVFIFPPSDEQLKSGTASVVCLLNNFYPREAKVQWKVDNALQSGNSQ 160
Rituximab_LC/1-213    80 EDAATYYCQQWTSNPPTFGGGTKLEIKRTVAAPSVFIFPPSDEQLKSGTASVVCLLNNFYPREAKVQWKVDNALQSGNSQ 159
Trastuzumab_LC/1-21   81 EDFATYYCQQHYTTPPTFGQGTKVEIKRTVAAPSVFIFPPSDEQLKSGTASVVCLLNNFYPREAKVQWKVDNALQSGNSQ 160

Bevacizumab_LC/1-21  161 ESVTEQDSKDSTYSLSSTLTLSKADYEKHKVYACEVTHQGLSSPVTKSFNRGEC 214
Adalimumab_LC/1-214  161 ESVTEQDSKDSTYSLSSTLTLSKADYEKHKVYACEVTHQGLSSPVTKSFNRGEC 214
Rituximab_LC/1-213   160 ESVTEQDSKDSTYSLSSTLTLSKADYEKHKVYACEVTHQGLSSPVTKSFNRGEC 213
Trastuzumab_LC/1-21  161 ESVTEQDSKDSTYSLSSTLTLSKADYEKHKVYACEVTHQGLSSPVTKSFNRGEC 214
```

Figure S2.1: 4AB Light Chain Alignment

```
Adalimumab_HC/1-450    1 EVQLVESGGGLVQPGRSLRLSCAASGFTFDDYAMHWVRQAPGKGLEWVSAITWNSGHIDYADSVEGRFTISRDNAKNSLY  80
Bevacizumab_HC/1-45    1 EVQLVESGGGLVQPGGSLRLSCAASGYTFTNYGMNWVRQAPGKGLEWVGWINTYTGEPTYAADFKRRFTFSLDTSKSTAY  80
Rituximab_HC/1-450     1 QVQLQQPGAELVKPGASVKMSCKASGYTFTSYNMHWVKQTPGRGLEWIGAIYPGNGDTSYNQKFKGKATLTADKSSSTAY  80
Trastuzumab_HC/1-44    1 EVQLVESGGGLVQPGGSLRLSCAASGFNIKDTYIHWVRQAPGKGLEWVARIYPTNGYTRYADSVKGRFTISADTSKNTAY  80

Adalimumab_HC/1-450   81 LQMNSLRAEDTAVYYCAKVSYL--STASSLDYWGQGTLVTVSSASTKGPSVFPLAPSSKSTSGGTAALGCLVKDYFPEPV 158
Bevacizumab_HC/1-45   81 LQMNSLRAEDTAVYYCAKYPHYYGSSHWYFDVWGQGTLVTVSSASTKGPSVFPLAPSSKSTSGGTAALGCLVKDYFPEPV 160
Rituximab_HC/1-450    81 MQLSSLTSEDSAVYYCARSTYY--GGDWYFNVWGAGTTVTVSAASTKGPSVFPLAPSSKSTSGGTAALGCLVKDYFPEPV 158
Trastuzumab_HC/1-44   81 LQMNSLRAEDTAVYYCSRWGGD---GFYAMDYWGQGTLVTVSSASTKGPSVFPLAPSSKSTSGGTAALGCLVKDYFPEPV 157

Adalimumab_HC/1-450  159 TVSWNSGALTSGVHTFPAVLQSSGLYSLSSVVTVPSSSLGTQTYICNVNHKPSNTKVDKKVEPKSCDKTHTCPPCPAPEL 238
Bevacizumab_HC/1-45  161 TVSWNSGALTSGVHTFPAVLQSSGLYSLSSVVTVPSSSLGTQTYICNVNHKPSNTKVDKKVEPKSCDKTHTCPPCPAPEL 240
Rituximab_HC/1-450   159 TVSWNSGALTSGVHTFPAVLQSSGLYSLSSVVTVPSSSLGTQTYICNVNHKPSNTKVDKKAEPKSCDKTHTCPPCPAPEL 238
Trastuzumab_HC/1-44  158 TVSWNSGALTSGVHTFPAVLQSSGLYSLSSVVTVPSSSLGTQTYICNVNHKPSNTKVDKKVEPKSCDKTHTCPPCPAPEL 237

Adalimumab_HC/1-450  239 LGGPSVFLFPPKPKDTLMISRTPEVTCVVVDVSHEDPEVKFNWYVDGVEVHNAKTKPREEQYNSTYRVVSVLTVLHQDWL 318
Bevacizumab_HC/1-45  241 LGGPSVFLFPPKPKDTLMISRTPEVTCVVVDVSHEDPEVKFNWYVDGVEVHNAKTKPREEQYNSTYRVVSVLTVLHQDWL 320
Rituximab_HC/1-450   239 LGGPSVFLFPPKPKDTLMISRTPEVTCVVVDVSHEDPEVKFNWYVDGVEVHNAKTKPREEQYNSTYRVVSVLTVLHQDWL 318
Trastuzumab_HC/1-44  238 LGGPSVFLFPPKPKDTLMISRTPEVTCVVVDVSHEDPEVKFNWYVDGVEVHNAKTKPREEQYNSTYRVVSVLTVLHQDWL 317

Adalimumab_HC/1-450  319 NGKEYKCKVSNKALPAPIEKTISKAKGQPREPQVYTLPPSRDELTKNQVSLTCLVKGFYPSDIAVEWESNGQPENNYKTT 398
Bevacizumab_HC/1-45  321 NGKEYKCKVSNKALPAPIEKTISKAKGQPREPQVYTLPPSREEMTKNQVSLTCLVKGFYPSDIAVEWESNGQPENNYKTT 400
Rituximab_HC/1-450   319 NGKEYKCKVSNKALPAPIEKTISKAKGQPREPQVYTLPPSRDELTKNQVSLTCLVKGFYPSDIAVEWESNGQPENNYKTT 398
Trastuzumab_HC/1-44  318 NGKEYKCKVSNKALPAPIEKTISKAKGQPREPQVYTLPPSREEMTKNQVSLTCLVKGFYPSDIAVEWESNGQPENNYKTT 397

Adalimumab_HC/1-450  399 PPVLDSDGSFFLYSKLTVDKSRWQQGNVFSCSVMHEALHNHYTQKSLSLSPG 450
Bevacizumab_HC/1-45  401 PPVLDSDGSFFLYSKLTVDKSRWQQGNVFSCSVMHEALHNHYTQKSLSLSPG 452
Rituximab_HC/1-450   399 PPVLDSDGSFFLYSKLTVDKSRWQQGNVFSCSVMHEALHNHYTQKSLSLSPG 450
Trastuzumab_HC/1-44  398 PPVLDSDGSFFLYSKLTVDKSRWQQGNVFSCSVMHEALHNHYTQKSLSLSPG 449
```

Figure S2.2: 4AB Heavy Chain Alignment

S3 Mass Spectrometry Instrumentation and Data Collection Details

S3.1 LC-MS intact mass analysis

For all three samples: deglycosylated whole, reduced and subunits, LC-MS samples were run on a Thermo Scientific Orbitrap Fusion Lumos, equipped with a heated electrospray ionization source (H-ESI) in positive ion mode with a Thermo Fisher Ultimate 3000 RSLCnano HPLC System. On H-ESI source, sheath gas was set to 30 arbitrary units (arb), and auxiliary gas was set to 10 arb. The ion transfer tube was set at 320 °Cand the vaporizer temperature was at 100°C. The sample were separated on a MAbPac RP, 4μM, 3.0×100 mm analytical column, held at 60°C.

The protein was eluted at a rate of 200 μL/min for a 10-minute gradient, where 0-7 minutes: 10% - 70% acetonitrile + 0.1% formic acid; 7-8.2 minutes: 95% acetonitrile + 0.1% formic acid, 8.2-10 minutes: 20% acetonitrile + 0.1% formic acid. MS spectra were acquired using full scans at 7500 resolution in the orbitrap within a range of 400-4000 m/z. The maximum injection time was set to 100 ms with an AGC target of 800000. Ten micro scans were employed, the RF lens was set to 60%, and a source energy of 75 V was applied.

S3.2 Middle-down LC-MS/MS analysis

LC–MS/MS analysis was performed using an U3000 RSLCnano system (Thermo Scientific, San Jose, CA) coupled to an Orbitrap Eclipse^{TM} Tribrid mass spectrometer (Thermo Scientific, San Jose, CA) fitted with a H-ESI source. About 1 μg of LC, Fc and Fd were separated with a gradient of 0.1% formic acid in acetonitrile (ACN) at 200 μL/min, from 25% to 65% in 10 min. For all experiments spray voltage was set to 3.8 kV, sheath gas settings was 20, auxiliary gas settings was 10, sweep gas settings was 0, vaporizer temperature was 100°C, ion transfer tube temperature was 320°C, and source fragmentation energy was 35 V. A first LC–MS experiment was acquired at 120,000 resolving power (at m/z 200) with a scan range set to m/z 1000–1600, an automatic gain control (AGC) target value of 4×105, and a maximum injection time of 246 ms. Fragmentation data were recorded using DDA LC–MS/MS experiments between 0 and 15 min for the antibody LC, Fc/2 and Fd. MS/MS scans were acquired at 120,000 resolving power (at m/z 200), an AGC target value of 1×105, and maximum injection time of 500 ms. All experiments were conducted using the Intact Protein mode with a pressure set to 1 mTorr in the ion-routing multipole (IRM).

S3.3 Bottom-up LC-MS/MS analysis

Liquid chromatography was performed using an Ultimate 3000 chromatography system (ThermoFisher, Massachusetts, USA) and a constant flow of 0.25 μl/min and a 15 cm reversed-phased column with a 75 μm inner diameter filled with Reprosil C18 (PepSep, Bruker, Germany). Mobile phase A was 0.1% formic acid and mobile phase B was 99.9% acetonitrile, 0.1% formic acid. The separation was carried out over 40 minutes as follows: linearly 3% to 32% phase B over 27 minutes, 32% to 50% phase B over 5 minutes, 50% to 100% phase B over 1 minute and held constant for 4 minutes. Then the B percentage was set back to 3% in the final 3 minutes.

MS/MS data acquired on a Thermo Orbitrap Eclipse for each sample were carried out in data-dependent acquisition mode with a cycle time of two seconds. In the first round, MS1 scan data were obtained at 60,000 resolution (at 400 m/z) with a mass range of 350–1,500 m/z. The automatic gain control (AGC) was set to 400000, with a maximum ion injection time of 50 ms. The radio frequency (RF) lens was set to 40%. The charge state filter was set to 1-10 and the dynamic exclusion was set to 30 s. Isolation for MS2 scans was performed in the quadrupole, with an isolation window of 1.6 Da. MS2 scan data were acquired at a resolution of 15,000 in the orbitrap, with an AGC target of 50000 and a maximum ion injection time of 22 ms. Higher energy collisional dissociation (HCD; stepped collision energy: 15, 30, and 45%) or electron-transfer dissociation (EThcD) was used for generating MS2 spectra, with the number of microscans set to 1.

S4 Intact Deconvolution Algorithm Details

This section extends the description our intact deconvolution algorithm (briefly described in the main manuscript) in more detail. As mentioned before, our intact deconvolution for each intact sample contains three major parts: merging spectra in a sliding window, deconvolution for each merged spectrum, and combining results for all spectra. Figure S4.1 provides a detailed illustration for each part.

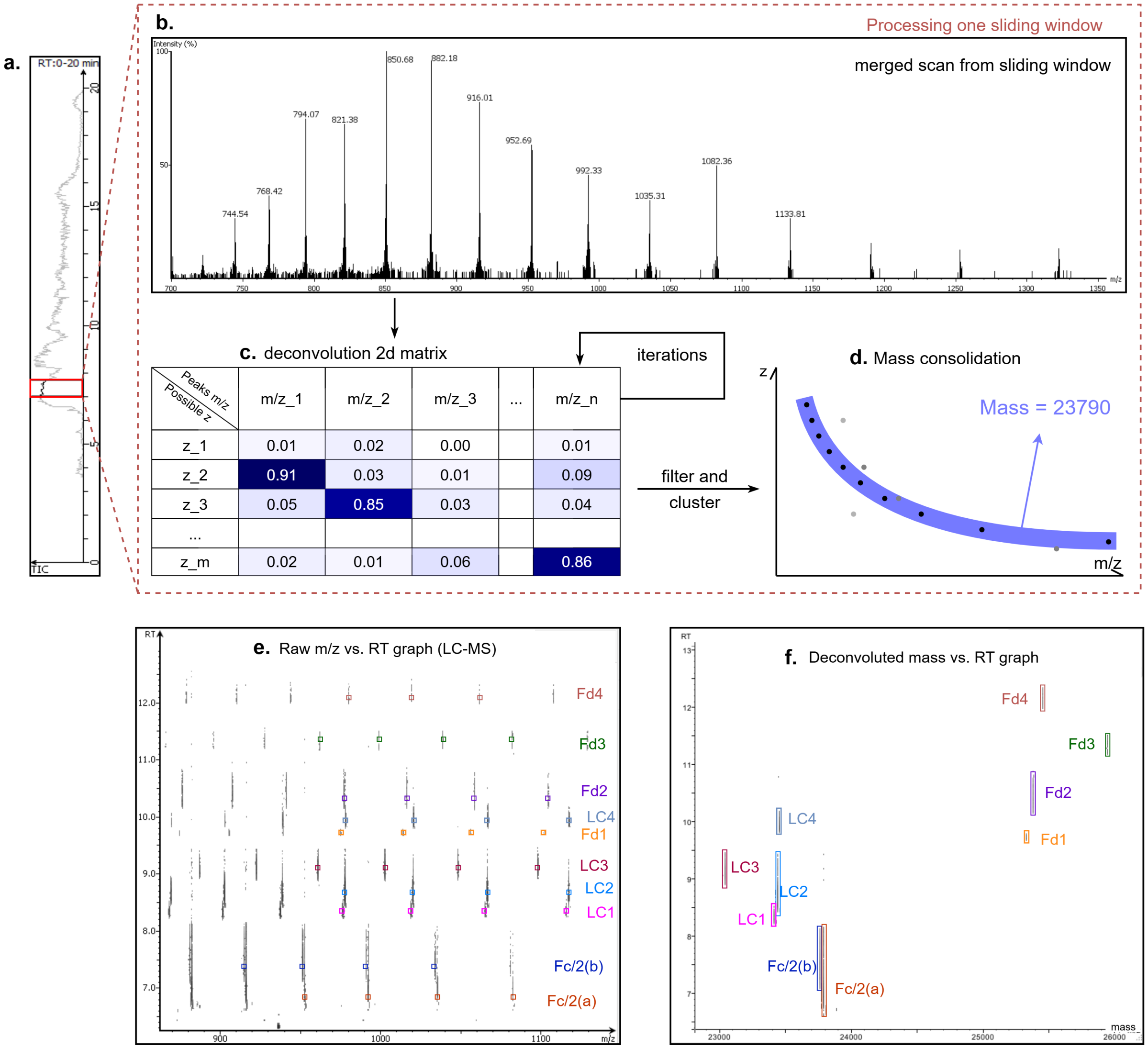

Figure S4.1: Feature-based intact deconvolution workflow. **a.** Total ion chromatograph (TIC) with selected sliding window in red. **b.** Merged input MS1 spectrum from the sliding window. **c.** 2D matrix with peaks m/z and possible charge states for deconvolution. **d.** Mass consolidation from deconvoluted 2D matrix, requiring consecutive charge states to support a reported mass. **e.** Raw LC-MS view of m/z vs. RT, with major features highlighted. **f.** Mass vs. RT view of deconvoluted masses.

Our algorithm includes two branches: isotopically resolved deconvolution and isotopically unresolved deconvolution. For all of our experiments, we collected low resolution (30k) LC-MS for the whole (mass range 120k to 160k) and reduced (mass range 20k to 50k) intact samples, making them isotopically unresolved, and high resolution (120k) data for the subunit samples (mass range 20k to 30k), making them isotopically resolved. Certain steps or

parameters in our deconvolution algorithm are the same for isotopically resolved and unresolved branches, while others are different for the two.

First, the sliding window approach (Figure S4.1a and b) allows for spectra from two different proteoforms that are RT separable to be separately deconvoluted. By adjusting the window size, the sliding window approach enhances signal to noise ratio by merging neighbouring spectra. Particularly, setting the sliding window size to 0 means to deconvolute each spectra separately without any merging, whereas setting it to the gradient length is equivalent to merge every spectra and deconvolute once. Our default window size was set at 0.1 minutes for isotopically unresolved samples and 0 for isotopically resolved samples, but can be adjusted according to the experiment and data.

Secondly, we deconvoluted each merged spectrum via charge assignment and inter-charge clustering. We converted each spectrum with n peaks into an 2D matrix with dimension $m \times n$, where m is the number of possible charges (Figure S4.1c). By default, we set possible charges in the range of 10 to 85. The matrix was initialized such that for each peak m/z, the values across charges were evenly distributed. Then, using a Bayesian deconvolution method outlined from UniDEC, we iteratively updated the matrix until convergence or until a maximum number of steps are reached.

For each charge, we filtered and collected local maximums as potential mass candidates. For isotopically unresolved data, we filtered local maximums with at least 10 % relative intensity. The local maxima represent average masses in different charges. For isotopically resolved data, besides the intensity filter, we also fit the isotope shape with a theoretical distribution to obtain the most abundant isotope, requiring a cosine similarity of at least 0.6. From the most abundant isotope m/z, we then calculated the monoisotopic m/z from the theoretical distribution.

Then, we consolidate the masses across charges, requiring at least 6 consecutive charges to support a reported mass (Figure S4.1d). The mass error tolerance for clustering masses is taken from the user input. For our experiments, we selected to use 100ppm, 30ppm, and 20ppm respectively for our whole, reduced, and subunit data. Eventually for the second step, we reported a list of masses (average masses for isotopically unresolved and monoisotopic masses for isotopically resolved), each associated to a list of differently charged m/z values (envelops).

Thirdly, having obtained a list of confident masses and corresponding charge envelops from each merged spectrum for each sliding window, we combine the results along the RT dimension to build "mass trails", which are similar masses within error tolerance (taken from user input) along a continuous range of RT, sharing overlapping supporting charge states. Each mass trail represents a proteoform, and is associated to a list of supporting "features", which are windows of m/z and RT, representing the proteoform at a particular charge.

An important note is that although we use the monoisotopic mass for isotopically resolved data during intact mass deconvolution and internally for sequencing, we still calculate the average mass from the monoisotopic mass and use it for grouping with isotopically unresolved intact samples, as well as for result display, in order to be consistent across tables.

As for calculating the intensity or abundance of each mass, the abundance of each deconvoluted mass within each merged spectra are calculated, and then when forming mass trails along the RT dimension, the weighted sum are taken the abundance of each proteoform mass. Firstly, the 2D matrix for Bayesian deconvolution keeps the relative intensity of the input peaks, so when extrapolating the masses from the m/z by charge table, the corresponding intensities are kept as deconvoluted mass relative intensities. For each merged spectra, the relative intensities of deconvoluted masses are multiplied by the base peak intensity of the middle spectrum in the sliding window. When building mass trails, a mass trail's intensity is the sum of intensities of the mass from each spectra along the trail.

S5 Inclusion List for Targeted Middle-Down

S5.1 HB-95 Experiment

Compound	Neutral Mass	m/z	charge	Apex RT (min)
LC1	23638.69	910.25	26	6.16
		946.58	25	
		985.90	24	
		1028.71	23	
LC2	23867.92	918.95	26	8.24
		955.71	25	
		995.49	24	
		1038.81	23	
Fd	24616.36	912.79	27	11.53
		947.83	26	
		985.61	25	
		1026.73	24	
Fc	24754.36	826.12	30	10.32
		854.57	29	
		885.09	28	
		917.84	27	

Table S5.1: Precursor Inclusion List of HB-95 Subunits for Targeted Middle-Down. Four LC-MS/MS raw files are generated, each targeting four precursors – one from each compound.

S5.2 Four mAb Mixture

Compound	Neutral Mass	m/z	charge	Apex RT (min)
Fc/2(a)	23791.52	850.68	28	6.93
		882.18	27	
		916.04	26	
		952.64	25	
Fc/2(b)	23759.63	849.54	28	7.26
		880.97	27	
		951.36	25	
		820.24	29	
LC1	23411.85	976.49	24	8.29
		901.45	26	
		937.47	25	
		868.10	27	
LC2	23443.75	938.71	25	8.55
		977.69	24	
		1066.53	22	
		1020.33	23	
LC3	23039.26	1002.67	23	9.02
		1048.20	22	
		960.97	24	
		1098.17	21	
LC4	23451.05	1117.79	21	9.87
		1020.59	23	
		978.06	24	
		1066.88	22	
Fd1	25328.32	1152.25	22	9.70
		1014.18	25	
		939.02	27	
		1056.35	24	
Fd2	25383.27	1016.27	25	10.32
		941.10	27	
		907.42	28	
		1154.84	22	
Fd3	25945.31	1180.36	22	11.35
		998.87	26	
		1082.08	24	
		895.79	29	
Fd4	25458.32	1019.19	25	12.05
		980.18	26	
		1061.56	24	
		910.27	28	

Table S5.2: Precursor Inclusion List of 4AB Mixture Subunits for Targeted Middle-Down. Four LC-MS/MS raw files are generated, each targeting ten precursors – one from each compound.

S6 Determining Sequence Start End for *de novo* Peptides

The following paragraphs describe in detail the last paragraph of section "Bottom-Up Peptide *de novo* Sequencing and Processing" in Methods, from data acquisition (S6.1), to model structure (S6.2), and to training and testing details with results (S6.3).

S6.1 Data Acquisition from IMGT

Whole-chain antibody sequences are obtained from the IMGT website https://www.imgt.org/3Dstructure-DB. "Domains and sequence alignment" was selected in the section **Display results**. In the section **Search using IMGT-ONTOLOGY concepts**-IDENTIFICATION, *IMGT receptor type* was set to "IG". For the field *IMGT chain description* in DESCRIPTION, several descriptions were chosen each time, outlined in Table S6.1 below, and the results were combined. After clicking **SEARCH**, the resulting multiple sequence alignment page is copied into a spreadsheet for further processing.

Chain	IMGT chain description	# Entries Found	# Sequences after processing
HC	H-GAMMA-1	931	1058
	H-GAMMA-2	73	
	H-GAMMA-3	1	
	H-GAMMA-4	252	
	H-ALPHA	1	
	H-DELTA	2	
	H-MU	7	
LC	L-KAPPA	6361	3218
	L-LAMBDA	1626	

Table S6.1: Table of IMGT Search Descriptions with Entries Found and Entries After Processing

The IMGT website alignment results is separated by domains. For example, the H-GAMMA results had separate alignments for the domains V, HINGE, CH1, CH2, and CH3. During processing, we join the sequences by their IMGT chain ID. If a V or C domain was missing, we would discard the sequence entry. We removed characters for alignment (. and #) and any spaces. We then removed duplicates of sequences. Eventually, we obtained 1058 heavy chains and 3218 light chains.

S6.2 Model Details

We modified the ESM model from their github repository https://github.com/facebookresearch/esm. We used the built-in alphabet "ESM-1b" and the model "ESM2" with 6 transformer layers and hidden dimension of 320. The model input was peptides, with a <cls> token prepended to the front and an <eos> token appended to the end. Each peptide has a label as the expected output, which is an integer value in the range 0 to 4, representing one of LC-START, LC-END, HC-START, HC-END, and OTHER. The model outputs the logits of the final layer after a Roberta head, with the same dimension as the input. We took the logits at the first position (corresponding to the <cls> token) and used Cross Entropy loss to compare it with the label.

S6.3 Train and Test Details

The heavy and light chains were both partitioned into train, validation, and test datasets with the ratio 7:1:2 and then for each of the three sets, the heavy and light chains were combined. For each light chain, we sampled 5 starting peptides (starting from the first position, with random lengths in the range 8 to 45), 5 ending peptides, and 5 middle peptides (where the starting position was randomly selected such that the peptide did not appear in the first or last 10 residues). For heavy chains, 15 peptides were randomly sampled for each position, so that we had relatively similar amounts of heavy and light peptides.

The model was trained on NVIDIA GeForce RTX 3060 GPU for 20 epochs (about 10,000 steps). Training was terminated when the validation loss stopped decreasing. For evaluation, the predicted classification is derived from

the highest logit of the `<cls>` token. We counted the total number of peptides as well as the number of peptides where the model predicted the label correctly. Eventually, the model evaluated with 99.7% accuracy.

We used this model to obtain sets of LC-START, LC-END, Fd-START, and Fc/2-END peptides. As for Fd-END and Fc/2-START, because the proteases IdeS and SpeB are both highly specific with strong motifs, we simply searched for the existence (or partial existence) of the cleaving motifs in the peptide. For example, in experiments where the IdeS (cleavage site LLG|G) was used to cleave the heavy chain, peptides ending with "LLGG" were considered Fd-END peptides, and peptides starting with the same motif are considered Fc/2-START peptides.

During assembly, the classified peptides are used to initiate the sequencing. For example, when sequencing for a subunit mass of 23000 Da for the subunit type of Fd, then we use the set of Fd-START peptides to initiate the DP table, and continue to assemble paths by extending right. For the other direction, we use the set of Fd-END peptides to initiate the DP table, and continue to assemble paths by extending left.

S7 Sequencing Example with Intermediary Step Results

Below is an example of sequencing Adalimumab Light chain from the 4 mAb mixture experiment. For the monoisotopic subunit intact mass 23397.6 Da and its corresponding targeted middle down data, we display intermediary results in constructing an LC sequence.

S7.1 Sequencing Right Intermediate Step Example

At mass index 39559 (corresponding to mass of 3955.9 Da), the bounded priority queue stored in the DP table stores the following paths:

Path	Middle-down matches	Unconfident residues	Score
ESGGGLVQPGGSLRVTLTCRASQDVNTAVSWYQQKPGK	2	5	32
MTQSPSLSASVGDRVVMTCRASQGLRNYLNWYQKK	2	5	32
SLSASVGDRVTLNNFYPREATLCRASQDVNTAVAWY	5	2	38
DLQMTQSPSSLSASVGDRVTLTCRASQGLRNYLAWY	13	0	48

Table S7.1: Example of paths at mass index 39559 with number of middle-down matches and unconfident residues, ranked by path score from low to high.

At the same mass index, all paths should have the same "base score", which is the average number of amino acids for the mass. At index 39559, the base score is 35. Then for the first path, the total score is calculated by 2 + 35 - 5 = 32, which is the number of middle-down matches plus the adjusted number of confident residues. The reason we do not use the path's actual length but the average length, is to not introduce bias for longer paths (e.g. preferring GG over N for all instances).

From the highest scoring path (starting with "DIQ"), we collect the possible extension candidates from the overlap graph. For each candidate, we then calculate the corresponding mass index and score.

NYLAWY (start peptide)	Mass index	Midddle-down matches	Unconfident residues	Score
YLAWYQQTPVR	46653	15	2	54
LAWYQQKPGNKPK	49615	16	1	59
LAWYQQKPGKAPK	49185	19	0	63

Table S7.2: Example of extended candidates from previously highest scoring path.

S7.2 Sequencing Left and Right Results

Top 10 sequences from extending right are saved, and same for extending left. Below lists the top results from extending left to right along with the scores.

1. DLQMTQSPSSLSASVGDRVTTLCRASQGLRNYLAWYQQKPGKAPKLLLYAASTLQSGVPSRFSGSGSGTDFTLTLSSLQPEDVATYY CAGRYNRAPYTFGQGTKVELKRTVAAPSVFLFPPSDEQLKSGTASVVCLLNNFYPREAKVQWKVDNALQSGNSQESVTEQDSKDSTY SLSSTLTLSKADYEKHKVYACEVTHQGLSSPVTKSFNRGEC, score = 282

2. DLQMTQSPSSLSASVGDRVTTLCRASQGLRNYLAWYQQKPGKAPQLLLYAASTLQSGVPSRFSGSGSGTDFTLTLSSLQPEDVATYY CQRYNRAPYTFGQGTKVELKRTVAAPSVFLFPPSDEQLKSGTASVVCLLNNFYPREAKVQWKVDNALQSGNSQESVTEQDSKDSTYS LSSTLTLSKADYEKHKVYACEVTHQGLSSPVTKSFNRGEC, score = 282

3. DLQMTQSPSSLSASVGDRVTTLCRASQGLRNYLAWYQQKPGKAPKLLLYAASTLQSGVPSRFSGSGSGTDFTLTLSSLQPEDVATYY CAGRYNRAPYTFGQGTKVELKRTVAAPSVFLFPPSDEQLKSGTASVVCLLNNFYPREAKVQWKVDNALQSGNSCESVTEQDSKDSTY DLSSTLTLSKADYEKHKVYACEVTHQGLSSPVTKSFNRGEC, score = 278

4. DLQMTQSPSSLSASVGDRVTTLCRASQGLRNYLAWYQQKPGKAPKLLLYAASTLQSGVPSRFSGSGSGTDFTLTLSSLQPEDVATYY CAGRYNRAPYTFGQGTKVELKRTVAAPSVFLFPPSDEQLKSGTASVVCLLNNFYPREAKVQWKVDNALQSGNSQESVTEQDSKDTSY SLSSTLTLSKADYEKHKVYACEVTHQGLSSPVTKSFNRGEC, score = 277

5. DLQMTQSPSSLSASVGDRVTTLCRASQGLRNYLAWYQQKPGKAPKLLLYAASTLQSGVPSRFSGSGSGTDFTLTLSSLQPEDVATYY
CAGRYNRAPYTFGQGTKVELKRTVAAPSVFLFPPSDEQLKSGTASVVCLLNNFYPREAKVQWKVDNALQSGNSQESVTEQDSKDTSY
SLSSTLTTVKADYEKHKVYACEVTHQGLSSPVTKSFNRGEC, score = 276
6. DLQMTQSPSSLSASVGDRVTTLCRASQGLRNYLAWYQQKPGKAPKLLLYAASTLQSGVPSRFSGSGSGTDFTLTLSSLQPEDVATYY
CAGRYNRAPYTFGQGTKVELKRTVAAPSVFLFPPSDEQLKSGTASVVCLLNNFYPREAKVQWKVDNALQSGNSQESVTEQDSQDTSY
SLSSTLTTVKADYEKHKVYACEVTHQGLSSPVTKSFNRGEC, score = 276
7. DLQMTQSPSSLSASVGDRVTTLCRASQGLRNYLAWYQQKPGKAPKLLLYAASTLQSGVPSRFSGSGSGTDFTLTLSSLQPEDVATYY
CAGRYNRAPYTFGQGTKVELKRTVAAPSVFLFPPSDEQLKSGTASVVCLLNNFYPREAKVQSVKVDNALQSGNSQESVTEQDSKDSTY
SLSSTLTLSKADYEKHKVYACEVTHQGLSSPVTKSFNRGEC, score = 275
8. DLQMTQSPSSLSASVGDRVTTLCRASQGLRNYLAWYQQKPGKAPKLLLYAASTLQSGVPSRFSGSGSGTDFTLTLSSLQPEDVATYY
CQRYNRAPYTFGAGGTKVELKRTVAAPSVFLFPPSDEQLKSGTASVVCLLNNFYPREAKVQSVKVDNALQSGNSQESVTEQDSKDSTY
SLSSTLTLSKADYEKHKVYACEVTHQGLSSPVTKSFNRGEC, score = 274
9. DLQMTQSPSSLSASVGDRVTTLCRASQGLRNYLAWYQQKPGKAPKLLLYAASTLQSGVPSRFSGSGSGTDFTLTLSSLQPEDVATYY
CAGRYNRAPYTFGAGGTKVELKRTVAAPSVFLFPPSDEQLKSGTASVVCLLNNFYPREAKVQWKVDNALQSGNSQESVTEQDSKDSTY
SLSSTLTLSKADYEKHKVYACEVTHQGLSSPVTKSFNRGEC, score = 274
10. DLQMTQSPSSLSASVGDRVTTLCRASQGLRNYLAWYQQKPGKAPKLLLYAASTLQSGVPSRFSGSGSGTDFTLTLSSLQPEDVATYY
CAGRYNRAPYTFGQGTKVELKRTVAAPSVFLFPPSDEQLKSGTASVVCLLNNFYPREAKVQWKVDNALQSGNSQESVTEQDSKDSTY
SLSSTLTLSKADYQKHKVYACEVTHEGLSSPVTKSFNRGEC, score = 271

Below are the top 10 sequences from extending right to left.

1. DLQMTQSPSSLLSASPGEKVTMTCRASQDVNTAVSWYQQKPGKAPKVLLYAASTLQSGVPSRFSGSGSGTDFTLTLSSLQPEDVATY
YCQRYNRAPYTFGQGTKVELKRTVAAPSVFLFPPSDEQLKSGTASVVCLLNNFYPREAKVQWKVDNALQSGNSQESVTEQDSKDSTY
SLSSTLTLSKADYEKHKVYACEVTHQGLSSPVTKSFNRGEC, score = 226
2. DLQMTQSPSSLSASVGDRVTLTCRASQGLRNYLAWYQQKPGKAPKLLLYAASTLQSGVPSRFSGSGSGTDFTLTLSSLQPEDVATYY
CQRYNRAPYTFGQGTKVELKRTVAAPSVFLFPPSDEQLKSGTASVVCLLNNFYPREAKVQWKVDNALQSGNSQESVTEQDSKDSTYS
LSSTLTLSKADYEKHKVYACEVTHQGLSSPVTKSFNRGEC, score = 226
3. DLQMTQSPSSLLSASPGEKVTMTCRASQDVNTAVAWYQQKPGKAPKLLLYAASTLQSGVPSRFSGSGSGTDFTLTLSSLQPEDVATY
YCQRYNRAPYTFGQGTKVELKRTVAAPSVFLFPPSDEQLKSGTASVVCLLNNFYPREAKVQWKVDNALQSGNSQESVTEQDSKDSTY
SLSSTLTLSKADYEKHKVYACEVTHQGLSSPVTKSFNRGEC, score = 225
4. DLQMTQSPSSLSASVGDRVTLTCRASQGLRNYLAWYQQKPGKAPKLLLYAASTLQSGVPSRFSGSGSGTDFTLTLSSLQPEDVATYY
CQRYNRAPYTFGQGTKVELKRTVAAPSVFLFPPSDEQLKSGTASVVCLLNNFYPREAKVQWKVDNALQSGNSQESVTEQDSKDSTYS
LSSTLTLSKADYEKHKVYACEVTHQGLSSPVTKSFNRGEC, score = 225
5. DLQMTQSPSSLLSASPGEKVTMTCRASQDVNTAVAWYQQKPGKAPKLLLYAASDVQSGVPSRFSGSGSGTDFTLTLSSLQPEDVATY
YCQRYNRAPYTFGQGTKVELKRTVAAPSVFLFPPSDEQLKSGTASVVCLLNNFYPREAKVQWKVDNALQSGNSQESVTEQDSKDSTY
SLSSTLTLSKADYEKHKVYACEVTHQGLSSPVTKSFNRGEC, score = 225
6. DLQMTQSPSSLSASPGEKVTKTCRASQGLRNYLAWYQQKPGKAPKLLLYAASTLQSGVPSRFSGSGSGTDFTLTLSSLQPEDVATYY
CQRYNRAPYTFGQGTKVELKRTVAAPSVFLFPPSDEQLKSGTASVVCLLNNFYPREAKVQWKVDNALQSGNSQESVTEQDSKDSTYS
LSSTLTLSKADYEKHKVYACEVTHQGLSSPVTKSFNRGEC, score = 224
7. DLQMTQSPSSLLSASPGEKVTMTCRASQDVNTAVAWYQQKPGKAPKLLLYAASTLQSGVPSRFSGSGSGTDFTLTLSSLQPEDVATY
YCQRYNRAPYTFGQGTKVELKRTVAAPSVFLFPPSDEQLKSGTASVVCLLNNFYPREAKVQWKVDNALQSGNSQESVTEQDSKDSTY
SLSSTLTLSKADYEKHKVYACEVTHQGLSSPVTKSFNRGEC, score = 224
8. DLQMTQSPSSLSASVGDRVTLSCRASQGLRNYLSWYQQKPGKAPKLLLYAASTLQSGVPSRFSGSGSGTDFTLTLSSLQPEDVATYY
CQRYNRAPYTFGQGTKVELKRTVAAPSVFLFPPSDEQLKSGTASVVCLLNNFYPREAKVQWKVDNALQSGNSQESVTEQDSKDSTYS
LSSTLTLSKADYEKHKVYACEVTHQGLSSPVTKSFNRGEC, score = 224

9. `DLQMTQSPSSTVASVGDRVTLSCRASQGLRNYLSWYQQKPGKAPKLLLYAASTLQSGVPSRFSGSGSGTDFTLTLSSLQPEDVATYY CQRYNRAPYTFGQGTKVELKRTVAAPSVFLFPPSDEQLKSGTASVVCLLNNFYPREAKVQWKVDNALQSGNSQESVTEQDSKDSTYS LSSTLTLSKADYEKHKVYACEVTHQGLSSPVTKSFNRGEC, score = 224`

10. `DLQMTQSPSSLSLASPGEKVTMTCRASQDVNTAVAWYQQKPGKAPKLLLYAASTLQSGVPSRFSGSGSGTDFTLTLSSLQPEDVATY YCQRYNRAPYTFGQGTKVELKRTVAAPSVFLFPPSDEQLKSGTASVVCLLNNFYPREAKVQWKVDNALQSGNSQESVTEQDSKDSTY SLSSTLTLSKADYEKHKVYACEVTHQGLSSPVTKSFNRGEC, score = 224`

S7.3 Joining Left and Right Results

Following is the result of joining the extending left to right result 1 and extending right to left result 1:

```
best connection point 46 total middle-down matched = 96
forward path: DLQMTQSPSSLSASVGDRVTTLCRASQGLRNYLAWYQQKPGKAPKL
backward path: LLYAASTLQSGVPSRFSGSGSGTDFTLTLSSLQPEDVATYYCQRYNRAPYTFGQGTKVELKRTVAAPSVFLF
PPSDEQLKSGTASVVCLLNNFYPREAKVQWKVDNALQSGNSQESVTEQDSKDSTYSLSSTLTLSKADYEKHKVYACEVTHQGLSSPV
TKSFNRGEC
```

For simplicity, not all joining pairings ($10 \times 10 = 100$ in total) are listed. Even though the extending left and right candidates are distinct, many results are identical after merging both directions. Eventually, four paths are reported, all with 96 middle-down fragment matches. Differences compared to the first sequence are highlighted.

1. DLQMTQSPSSLSASVGDRVTLTCRASQGLRNYLAWYQQKPGKAPKLLLYAASTLQSGVPSRFSG SGSGTDFTLTLSSLQPEDVATYYCQRYNRAPYTFGQGTKVELKRTVAAPSVFLFPPSDEQLKSG TASVVCLLNNFYPREAKVQWKVDNALQSGNSQESVTEQDSKDSTYSLSSTLTLSKADYEKHKVY ACEVTHQGLSSPVTKSFNRGEC

2. DLQMTQSPSSLSASVGDRVTTLCRASQGLRNYLAWYQQKPGKAPKLLLYAASTLQSGVPSRFSG SGSGTDFTLTLSSLQPEDVATYYCQRYNRAPYTFGQGTKVELKRTVAAPSVFLFPPSDEQLKSG TASVVCLLNNFYPREAKVQWKVDNALQSGNSQESVTEQDSKDSTYSLSSTLTLSKADYEKHKVY ACEVTHQGLSSPVTKSFNRGEC

3. DLQMTQSPSSLSASVGDRVTLTCRASQGLRNYLAWYQQKPGKAPKLLLYAASTLKSGVPSRFSG SGSGTDFTLTLSSLQPEDVATYYCQRYNRAPYTFGQGTKVELKRTVAAPSVFLFPPSDEQLKSG TASVVCLLNNFYPREAKVQWKVDNALQSGNSQESVTEQDSKDSTYSLSSTLTLSKADYEKHKVY ACEVTHQGLSSPVTKSFNRGEC

4. DLQMTQSPSSLSASVGDRVTTLCRASQGLRNYLAWYQQKPGKAPKLLLYAASTLKSGVPSRFSG SGSGTDFTLTLSSLQPEDVATYYCQRYNRAPYTFGQGTKVELKRTVAAPSVFLFPPSDEQLKSG TASVVCLLNNFYPREAKVQWKVDNALQSGNSQESVTEQDSKDSTYSLSSTLTLSKADYEKHKVY ACEVTHQGLSSPVTKSFNRGEC

S7.4 SPIDER Calibration Results

Consider the second sequence from joining left and right results above. The bottom-up path scorer using *de novo* PSMs highlight the red-colored region (...RVTTLCRA...) as unconfident. We then collect SPIDER peptides that overlap with the unconfident region, such that the total mass are exactly equal. 5 SPIDER peptides support the region to be "TL" while 37 others support "LT". Therefore, after SPIDER calibration of the four resulting sequencing from the previous step, and after removing duplicate sequences, only one sequencing result remains (exactly the same as the first result from joining left and right).

S7.5 Ile/Leu Determination Results

Table S7.3 below lists the Ile/Leu results from EThcD spectra statistics for every position.

Region	Position	#PSM with I	#PSM with L	Confidence	Prediction	True I/L
FR1	2	4	1	High	I	I
FR1	11	0	2	High	L	L
FR1	21	4	0	High	I	I
CDR1	29	2	0	High	I	I
CDR1	33	0	9	High	L	L
FR2	46	0	1	Medium	L	L
FR2	47	0	3	High	L	L
FR2	48	2	0	High	I	I
CDR2	54	0	6	High	L	L
FR3	73	0	6	High	L	L
FR3	75	1	0	Medium	I	I
FR3	78	0	0	Low	L	L
FR4	106	14	0	High	I	I
FR4	117	1	0	Medium	I	I
FR4	125	0	6	High	L	L
C	135	0	16	High	L	L
C	136	3	13	High	L	L
C	154	1	11	High	L	L
C	175	0	16	High	L	L
C	179	0	7	High	L	L
C	181	0	11	High	L	L
C	201	0	34	High	L	L

Table S7.3: EThcD Spectra Evidence for Every Position in Adalimumab Light Chain

S8 HB95 Supplementary Results

S8.1 Intact Mass Results

Mass	Intensity (%)	#z	Annotation	Expected Mass	Mass Error (ppm)
**146182.45**	**100**	**26**	**HC*2+LC1+LC2**	**146179.361**	**21.13**
**145952.61**	**89.96**	**31**	**HC*2+LC1*2**	**145950.076**	**17.36**
**146413.11**	**44.26**	**25**	**HC*2+LC2*2**	**146408.646**	**30.49**
147401.05	4.69	13	HC*2+LC1*2+G0F	147395.426	38.16
147793.89	4.66	14	HC*2+LC1+LC2+G1F	147786.854	47.61
145465.83	2.53	10			
146537.25	2.45	12	HC*2+LC2*2+K	146536.846	2.76

Table S8.1: Whole intact mass results with annotation. Masses are average masses in Da. and #z represents the number of charges to support the mass. The Expected Masses are calculated from constructed sequences, by calculating the average mass from residues of HC and LC, considering unreduced disulfide bonds. The annotated "HC" includes Lys. truncation and pyroglutamic acid from Gln at the N-terminal. The three expected antibody masses are reported within one order of magnitude. Three additional annotated masses and one unannotated mass are reported within two orders of magnitude. The additional masses are result of incomplete deglycosylation or Lys. truncation.

Mass	Intensity (%)	#z	Annotation	Expected Mass	Mass Error (ppm)
**49352.92**	**100**	**41**	**HC, 1S=S**	**49352.927**	**-0.14**
**23638.22**	**64.04**	**24**	**LC1**	**23639.139**	**-38.88**
**23867.37**	**48.30**	**27**	**LC2**	**23868.424**	**-44.16**
49335.53	27.17	25	HC-18		
49372.27	18.66	25	HC+19		
23622.25	16.41	21	LC1-16		
35603.89	13.99	29	Enzyme		

Table S8.2: Reduced intact mass results with annotation, within one order of magnitude. The entries with lesser intensity are hidden, as there are over 100 masses detected with above 1% intensity. The annotated "HC" includes Lyc truncation as well as pyroglutamic acid from Gln. We also considered one reformed (reoxidized) disulfide bond on the HC when calculating the expected mass.

Mm	Intensity %	#z	Annotation	Exp. Mm	Mm Error (ppm)	Ma
23624.3	100.0	24	LC1	23624.310	-0.42	23639.43
23853.5	43.13	23	LC2	23853.524	-1.01	23868.16
23602.3	34.71	24	Fd, PyroGlu	23602.311	-0.45	23617.23
23737.3	30.96	23	Fc/2 major site	23737.305	-0.20	23754.70
24255.1	9.60	22	Fc/2 minor site	24255.110	-0.41	24272.18
20219.1	7.06	18				
22660.2	6.28	24				

Table S8.3: Subunit intact mass results in monoisotopic mass (M_m), with annotation and mass error. The right-most column is the calculated average mass (M_a). Expected monoisotopic mass is calculated by the sum of residue monoisotopic masses in the sequence. Fd is annotated with PyroGlu. Fc/2 is annotated with Lyc. truncation. Fc/2 minor site is five AA downstream of Fc/2 major site. A total of 25 mass entries are reported above 1% relative intensity. Entries below 5% are omitted in this table.

S8.2 Constructed Sequences

```
>ab_hc
QVQLKQSGPGLVQPSQSLSLTCTVSGFSLTSYGVHWVRQPPGKGLEWLGVIWSGGSTDYNAAFISRLSIRKDNSKSQVFFKMNSLQADDTAL
YYCARTFTTSTSAWFAYWGQGTLVTVSAAKTTAPSVYPLAPVCGDTTGSSVTLGCLVKGYFPEPVTLTWNSGSLSSGVHTFPAVLQSDLYTL
SSSVTVTSSTWPSQSITCNVAHPASSTKVDKKIEPRGPTIKPCPPCKCPAPNLLGGPSVFIFPPKIKDVLMISLSPIVTCVVVDVSEDDPDV
QISWFVNNVEVHTAQTQTHREDYNSTLRVVSALPIQHQDWMSGKEFKCKVNNKDLPAPIERTISKPKGSVRAPQVYVLPPPEEEMTKKQVTL
TCMVTDFMPEDLYVEWTNDGKTELNYKNTEPVLDSDGSYFMYSKLRVEKKNWVERNSYSCSVVHEGLHNHHTTKSFSRTPG
>ab_lc1
NIVMTQSPKSMSMSVGERVTLTCKASENVVTYVSWYQQKPEQSPKLLIYGASNRYTGVPDRFTGSGSATDFTLTISSVQAEDLADYHCGQGY
SYPYTFGGGTKLEIKRADAAPTVSIFPPSSEQLTSGGASVVCFLNNFYPKDINVKWKIDGSERQNGVLNSWTDQDSKDSTYSMSSTLTLTKD
EYERHNSYTCEATHKTSTSPIVKSFNRNEC
>ab_lc2
SIVMTQTPKFLLVSAGDRVTITCKASQSVSNDVAWYQQKPGQSPKLLIYYASNRYTGVPDRFTGSGYGTDFTFTISTVQAEDLAVYFCQQDY
SSPPWTFGGGTKLEIRRADAAPTVSIFPPSSEQLTSGGASVVCFLNNFYPKDINVKWKIDGSERQNGVLNSWTDQDSKDSTYSMSSTLTLTK
DEYERHNSYTCEATHKTSTSPIVKSFNRNEC
```

The protease SpeB cleavage site is after the 231st residue, PPCK|CPAP. The minor cleavage site is after the 236th residue, PAPN|LLGG.

S8.3 Western Blot Result

Western blot analysis for HB-95 sequenced result validation Expressed recombinant antibodies from our constructed sequences were used to capture HLA-I complexes along with immunopeptides. HLA-I complexes were then separated from the immunopeptides using SDS-PAGE and transferred onto PVDF membranes. These membranes were then probed with anti-HLA-I antibodies (for secondary validation) and detected via chemiluminescent Western blotting. Strong bands corresponding to HLA-I heavy chains (around 44 kDa) were clearly visible, confirming successful immunoprecipitation (Supplementary Figure S8.1A). The Western blot results verified the enrichment of HLA-I complexes, as shown by distinct and intense bands representing the HLA-I heavy chains. Minimal nonspecific binding was observed, demonstrating the specificity of the HB-95 antibody.

As for the immunopeptides, LC-MS/MS analysis was further conducted on the eluted HLA-I complexes. Mass spectrometry data were acquired using a Nano-Elute high-performance liquid chromatography system coupled with a high-resolution mass spectrometer (Bruker timsTOF Pro2). The instrument was operated in DDA mode with a total run time of 60 minutes. The full mass scan range was m/z 100–4000, and the PASEF settings included 10 MS/MS scans per cycle, with a total cycle time of 2.22 seconds and an ion intensity threshold of 2500. Raw mass spectrometry files (.d) were generated during acquisition. All raw data were analyzed using the PEAKS DeepNovo Peptidome platform (Bioinformatics Solutions, Inc.) against SwissProt human protein database. This analysis combined de novo sequencing, database searches, and homology searches to identify peptides. The identified peptides displayed the characteristic length distribution typical of HLA-I peptides (Supplementary Figure S8.1B).

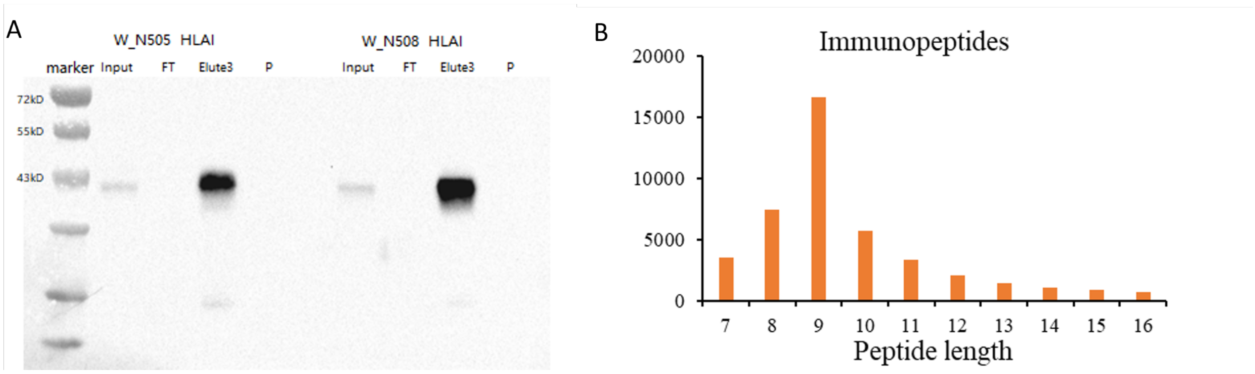

Figure S8.1: Western Blot (A) and immunopeptidome (B) results for the validation of *de novo* antibody sequences from the HB-95 sample.

S9 4AB Mixture Supplementary Results

S9.1 Intact Mass Results

Mass	Intensity (%)	#z	Annotation	Expected Mass	Mass Error (ppm)
**145166.20**	**100**	**27**	**TRA**	**145166.642**	**-3.05**
**146309.38**	**81.62**	**29**	**BEV**	**146308.018**	**9.31**
**145190.53**	**49.84**	**28**	**ADA**	**145190.988**	**-3.16**
**144187.91**	**41.32**	**29**	**RIT**	**144185.786**	**14.73**
145318.14	13.60	25	ADA + K	145319.188	-7.21
144314.89	11.11	27	RIT + K	144313.986	6.26
145269.05	10.36	21			
146410.50	10.35	20			
145331.98	5.48	20			
145445.19	4.97	24	ADA + 2K	145447.388	-15.11
148769.14	1.13	12			
145605.94	1.10	18			

Table S9.1: Whole intact mass results with annotation. Masses are average masses in Da. and #z represents the number of charges to support the mass. The expected masses are calculated from true mAb sequences. Lysine truncation is considered for all antibody heavy chains, unless otherwise annotated. Rituximab HC and LC both contain pyroglutamic acid from Gln on the N terminal.

Mass	Intensity (%)	#z	Annotation	Expected Mass	Mass Error (ppm)
**23442.11**	**100**	**20**	**TRA LC**	**23443.048**	**-40.01**
**23449.77**	**86.52**	**18**	**BEV LC, 1 S=S**	**23449.007**	**32.54**
**23038.50**	**59.45**	**19**	**RIT LC, PyroGlu, 1 S=S**	**23037.644**	**37.16**
**23410.93**	**50.72**	**22**	**ADA LC, 1 S=S**	**23410.113**	**34.90**
**49156.70**	**43.83**	**44**	**TRA HC**	**49156.398**	**6.14**
**49198.33**	**22.35**	**34**	**ADA HC**	**49199.492**	**-23.62**
**49068.73**	**21.79**	**27**	**RIT HC (PyroGlu)**	**49069.361**	**-12.85**
**49718.69**	**16.89**	**31**	**BEV HC**	**49719.113**	**-3.48**
23425.91	11.88	19	TRA LC - 16		
49110.20	7.70	27			
23397.94	7.68	17	ADA LC - 13		

Table S9.2: Reduced intact mass results above 5% intensity. The entries with lower intensities are hidden, as there are 43 entries above 1% intensity in total. The annotated “HC” all have Lysine truncation

M_m	Intensity %	#z	Annotation	Exp. M_m	M_m Error (ppm)	M_a
23775.9	100.0	26	BEV, TRA Fc/2	23775.919	-0.80	23791.52
23428.5	66.08	22	TRA LC	23428.513	-0.55	23443.75
23743.9	59.58	25	ADA, RIT Fc/2	23743.947	-1.98	23759.63
23397.6	28.25	25	ADA LC	23397.599	0.04	23411.85
25367.5	25.14	26	TRA Fd	25367.507	-0.25	25383.27
23025.3	27.30	23	RIT LC, PyroGlu	23025.331	-1.35	23039.26
23436.4	27.18	23	BEV LC	23436.423	-0.98	23451.05
23759.9	19.99	22	ADA, RIT Fc/2 + 16<br>BEV, TRA Fc/2 - 16			23775.00
25929.6	13.87	23	BEV Fd	25929.649	-1.89	25945.31
23793.9	12.49	23	BEV, TRA Fc/2 + 18			23808.53
25442.4	11.09	24	ADA Fd	25442.513	-4.44	25458.32
23418.4	9.26	16	BEV LC - 18			23437.10
23410.4	8.66	18	TRA LC - 18			23426.93
23725.9	8.14	19	ADA, RIT Fc/2 - 18			23742.03
23444.5	6.54	19	TRA LC + 16			23458.92
25349.5	5.87	22	TRA Fd - 18			25365.20
25312.3	5.25	25	RIT Fd, PyroGlu	25312.351	-2.13	25328.32

Table S9.3: Subunit intact mass results in monoisotopic mass (M_m), with annotation and mass error. The right-most column is the calculated average mass (M_a). Expected monoisotopic mass is calculated by the sum of residue monoisotopic mass in the sequence. RIT LC and Fd are annotated with PyroGlu. All Fc/2 expected masses include Lys. truncation. There are 11 reported masses above 10%, 17 above 5%, and 58 above 1% relative intensity. Results with lower intensities are omitted.

S9.2 Constructed Sequences and Alignment

```
Bevacizumab_LC     DIQMTQSPSSLSASVGDRVTITCSASQDISNYLNWYQQKPGKAPKVLIYFTSSLHSGVPS 60
constructed        DIQMTQSPSSLSASVGDRVTITCSASQDLSNYLNWYQQKPGKAPKVLIYFTSSLHSGVPS 60
                   ****************************:*******************************

Bevacizumab_LC     RFSGSGSGTDFTLTISSLQPEDFATYYCQQYSTVPWTFGQGTKVEIKRTVAAPSVFIFPP 120
constructed        RFSGSGSGTDFTLTISSLQPEDFATYYCQQYSTVPWTFGQGTKVEIKRTVAAPSVFIFPP 120
                   ************************************************************

Bevacizumab_LC     SDEQLKSGTASVVCLLNNFYPREAKVQWKVDNALQSGNSQESVTEQDSKDSTYSLSSTLT 180
constructed        SDEQLKSGTASVVCLLNNFYPREAKVQWKVDNALQSGNSQESVTEQDSKDSTYSLSSTLT 180
                   ************************************************************

Bevacizumab_LC     LSKADYEKHKVYACEVTHQGLSSPVTKSFNRGEC 214
constructed        LSKADYEKHKVYACEVTHQGLSSPVTKSFNRGEC 214
                   **********************************


Trastuzumab_LC     DIQMTQSPSSLSASVGDRVTITCRASQDVNTAVAWYQQKPGKAPKLLIYSASFLYSGVPS 60
constructed        DIQMTQSPSSLSASVGDRVTITCRASQDVNTAVAWYQQKPGKAPKLLIYSASFLYSGVPS 60
                   ************************************************************

Trastuzumab_LC     RFSGSRSGTDFTLTISSLQPEDFATYYCQQHYTTPPTFGQGTKVEIKR-TVAAPSVFIFP 119
constructed        RFSGSRSGTDFTLTISSLQPEDFATYYCQQHYTTPPTFGQGTKVEIKGVTVAAPSVFIFP 120
                   ***********************************************  ***********

Trastuzumab_LC     PSDEQLKSGTASVVCLLNNFYPREAKVQWKVDNALQSGNSQESVTEQDSKDSTYSLSSTL 179
constructed        PSDEQLKSGTASVVCLLNNFYPREAKVQWKVDNALQSGNSQESVTEQDSKDSTYSLSSTL 180
                   ************************************************************

Trastuzumab_LC     TLSKADYEKHKVYACEVTHQGLSSPVTKSFNRGEC 214
constructed        TLSKADYEKHKVYACEVTHQGLSSPVTKSFNRGEC 215
                   ***********************************


Adalimumab_LC     DIQMTQSPSSLSASVGDRVTITCRASQGIRNYLAWYQQKPGKAPKLLIYAASTLQSGVPS 60
constructed       DIQMTQSPSSLSASVGDRVTITCRASQGIRNYLAWYQQKPGKAPKLLIYAASTLQSGVPS 60
                  ************************************************************

Adalimumab_LC     RFSGSGSGTDFTLTISSLQPEDVATYYCQRYNRAPYTFGQGTKVEIKRTVAAPSVFIFPP 120
constructed       RFSGSGSGTDFTLTISSLQPEDVATYYCQRYNRAPYTFGQGTKVEIKRTVAAPSVFIFPP 120
                  ************************************************************

Adalimumab_LC     SDEQLKSGTASVVCLLNNFYPREAKVQWKVDNALQSGNSQESVTEQDSKDSTYSLSSTLT 180
constructed       SDEQLKSGTASVVCLLNNFYPREAKVQWKVDNALQSGNSQESVTEQDSKDSTYSLSSTLT 180
                  ************************************************************

Adalimumab_LC     LSKADYEKHKVYACEVTHQGLSSPVTKSFNRGEC 214
constructed       LSKADYEKHKVYACEVTHQGLSSPVTKSFNRGEC 214
                  **********************************


Rituximab_LC     QIVLSQSPAILSASPGEKVTMTCRASSSVSYIHWFQQKPGSSPKPWIYATSNLASGVPVR 60
```

```
constructed      QIVLSQSPAILSASPGEKVTMTCRASSSVSYIHWFQQKPGSSPKPWIYATSNLASGVPVR 60
                 ************************************************************

Rituximab_LC     FSGSGSGTSYSLTISRVEAEDAATYYCQQWTSNPPTFGGGTKLEIKRTVAAPSVFIFPPS 120
constructed      FSGSGSGTSYSLTISRVEAEDAATYYCQQWTSNPPTFGGGTKLEIKRTVAAPSVFIFPPS 120
                 ************************************************************

Rituximab_LC     DEQLKSGTASVVCLLNNFYPREAKVQWKVDNALQSGNSQESVTEQDSKDSTYSLSSTLTL 180
constructed      DEQLKSGTASVVCLLNNFYPREAKVQWKVDNALQSGNSQESVTEQDSKDSTYSLSSTLTL 180
                 ************************************************************

Rituximab_LC     SKADYEKHKVYACEVTHQGLSSPVTKSFNRGEC 213
constructed      SKADYEKHKVYACEVTHQGLSSPVTKSFNRGEC 213
                 *********************************


Bevacizumab_HC     EVQLVESGGGLVQPGGSLRLSCAASGYTFTNYGMNWVRQAPGKGLEWVGWINTYTGEPTY 60
constructed        EVQLVESGGGLVQPGGSLRLSCAASGYTFTNYGMNWVRQAPGKGLEWVGWINTYTGEPTY 60
                   ************************************************************

Bevacizumab_HC     AADFKRRFTFSLDTSKSTAYLQMNSLRAEDTAVYYCAKYPHYYGSSHWYFDVWGQGTLVT 120
constructed        AADFKRRFTFSLDTSKSTAYLQMNSLRAEDTAVYYCAKYPHYYGSSHWYFDVWGQGTLVS 120
                   ***********************************************************:

Bevacizumab_HC     VSSASTKGPSVFPLAPSSKSTSGGTAALGCLVKDYFPEPVTVSWNSGALTSGVHTFPAVL 180
constructed        LSSASTKGPSVFPLAPSSKSTSGGTAALGCLVKDYFPEPVTVSWNSGALTSGVHTFPAVL 180
                   :***********************************************************

Bevacizumab_HC     QSSGLYSLSSVVTVPSSSLGTQTYICNVNHKPSNTKVDKKVEPKSCDKTHTCPPCPAPEL 240
constructed        QSSGLYSLSSVVTVPSSSLGTQTYICNVNHKPSNTKVDKKVEPKSCDKTHTCPPCPAPEL 240
                   ************************************************************

Bevacizumab_HC     LGGPSVFLFPPKPKDTLMISRTPEVTCVVVDVSHEDPEVKFNWYVDGVEVHNAKTKPREE 300
constructed        LGGPSVFLFPPKPKDTLMISRTPEVTCVVVDVSHEDPEVKFNWYVDGVEVHNAKTKPREE 300
                   ************************************************************

Bevacizumab_HC     QYNSTYRVVSVLTVLHQDWLNGKEYKCKVSNKALPAPIEKTISKAKGQPREPQVYTLPPS 360
constructed        QYNSTYRVVSVLTVLHQDWLNGKEYKCKVSNKALPAPIEKTISKAKGQPREPQVYTLPPS 360
                   ************************************************************

Bevacizumab_HC     REEMTKNQVSLTCLVKGFYPSDIAVEWESNGQPENNYKTTPPVLDSDGSFFLYSKLTVDK 420
constructed        REEMTKNQVSLTCLVKGFYPSDIAVEWESNGQPENNYKTTPPVLDSDGSFFLYSKLTVDK 420
                   ************************************************************

Bevacizumab_HC     SRWQQGNVFSCSVMHEALHNHYTQKSLSLSPG 452
constructed        SRWQQGNVFSCSVMHEALHNHYTQKSLSLSPG 452
                   ********************************


Trastuzumab_HC     EVQLVESGGGLVQPGGSLRLSCAASGFNIKDTYIHWVRQAPGKGLEWVARIYPTNGYTRY 60
constructed        EVQLVESGGGLVQPGGSLRLSCAASGFNIKDTYIHWVRQAPGKGLEWVARIYPTNGYTRY 60
                   ************************************************************
```

```
Trastuzumab_HC      ADSVKGRFTISADTSKNTAYLQMNSLRAEDTAVYYCSRWGGDGFYAMDYWGQGTLVTVSS 120
constructed         ADSVKGRFTISADTSKNTAYLQMNSLRAEDTAVYYCSRWGGDGFYAMDYWGQGTLVTVSS 120
                    ************************************************************

Trastuzumab_HC      ASTKGPSVFPLAPSSKSTSGGTAALGCLVKDYFPEPVTVSWNSGALTSGVHTFPAVLQSS 180
constructed         ASTKGPSVFPLAPSSKSTSGGTAALGCLVKDYFPEPVTVSWNSGALTSGVHTFPAVLQSS 180
                    ************************************************************

Trastuzumab_HC      GLYSLSSVVTVPSSSLGTQTYICNVNHKPSNTKVDKKVEPKSCDKTHTCPPCPAPELLGG 240
constructed         GLYSLSSVVTVPSSSLGTQTYICNVNHKPSNTKVDKKVEPKSCDKTHTCPPCPAPELLGG 240
                    ************************************************************

Trastuzumab_HC      PSVFLFPPKPKDTLMISRTPEVTCVVVDVSHEDPEVKFNWYVDGVEVHNAKTKPREEQYN 300
constructed         PSVFLFPPKPKDTLMISRTPEVTCVVVDVSHEDPEVKFNWYVDGVEVHNAKTKPREEQYN 300
                    ************************************************************

Trastuzumab_HC      STYRVVSVLTVLHQDWLNGKEYKCKVSNKALPAPIEKTISKAKGQPREPQVYTLPPSREE 360
constructed         STYRVVSVLTVLHQDWLNGKEYKCKVSNKALPAPIEKTISKAKGQPREPQVYTLPPSREE 360
                    ************************************************************

Trastuzumab_HC      MTKNQVSLTCLVKGFYPSDIAVEWESNGQPENNYKTTPPVLDSDGSFFLYSKLTVDKSRW 420
constructed         MTKNQVSLTCLVKGFYPSDIAVEWESNGQPENNYKTTPPVLDSDGSFFLYSKLTVDKSRW 420
                    ************************************************************

Trastuzumab_HC      QQGNVFSCSVMHEALHNHYTQKSLSLSPG 449
constructed         QQGNVFSCSVMHEALHNHYTQKSLSLSPG 449
                    *****************************


Adalimumab_HC     EVQLVESGGGLVQPGRSLRLSCAASGFTFDDYAMHWVRQAPGKGLEWVSAITWNSGHIDY 60
constructed       EVQLVESGGGLVQPGRSLRLSCAASGFTFDDYAMHWVRQAPGKGLEWVSAITWNSGHIDY 60
                  ************************************************************

Adalimumab_HC     ADSVEGRFTISRDNAKNSLYLQMNSLRAEDTAVYYCAKVSYLSTASSLDYWGQGTLVTVS 120
constructed       ADSVEGRFTISRDNAKNSLYLQMNSLRAEDTAVYYCAKVSYLSTASSLDYWGQGTLVTVS 120
                  ************************************************************

Adalimumab_HC     SASTKGPSVFPLAPSSKSTSGGTAALGCLVKDYFPEPVTVSWNSGALTSGVHTFPAVLQS 180
constructed       SASTKGPSVFPLAPSSKSTSGGTAALGCLVKDYFPEPVTVSWNSGALTSGVHTFPAVLQS 180
                  ************************************************************

Adalimumab_HC     SGLYSLSSVVTVPSSSLGTQTYICNVNHKPSNTKVDKKVEPKSCDKTHTCPPCPAPELLG 240
constructed       SGLYSLSSVVTVPSSSLGTQTYICNVNHKPSNTKVDKKVEPKSCDKTHTCPPCPAPELLG 240
                  ************************************************************

Adalimumab_HC     GPSVFLFPPKPKDTLMISRTPEVTCVVVDVSHEDPEVKFNWYVDGVEVHNAKTKPREEQY 300
constructed       GPSVFLFPPKPKDTLMISRTPEVTCVVVDVSHEDPEVKFNWYVDGVEVHNAKTKPREEQY 300
                  ************************************************************

Adalimumab_HC     NSTYRVVSVLTVLHQDWLNGKEYKCKVSNKALPAPIEKTISKAKGQPREPQVYTLPPSRD 360
constructed       NSTYRVVSVLTVLHQDWLNGKEYKCKVSNKALPAPIEKTISKAKGQPREPQVYTLPPSRD 360
                  ************************************************************
```

```
Adalimumab_HC      ELTKNQVSLTCLVKGFYPSDIAVEWESNGQPENNYKTTPPVLDSDGSFFLYSKLTVDKSR 420
constructed        ELTKNQVSLTCLVKGFYPSDIAVEWESNGQPENNYKTTPPVLDSDGSFFLYSKLTVDKSR 420
                   ************************************************************

Adalimumab_HC      WQQGNVFSCSVMHEALHNHYTQKSLSLSPG 450
constructed        WQQGNVFSCSVMHEALHNHYTQKSLSLSPG 450
                   ******************************


Rituximab_HC      QVQLQQPGAELVKPGASVKMSCKASGYTFTSYNMHWVKQTPGRGLEWIGAIYPGNGDTSY 60
constructed       QVQLQQPGAELVKPGASVKMSCKASGYTFTSYNMHWVKQTPGRGLEWIGAIYPGNGDTSY 60
                  ************************************************************

Rituximab_HC      NQKFKGKATLTADKSSSTAYMQLSSLTSEDSAVYYCARSTYYGGDWYFNVWGAGTTVTVS 120
constructed       NQKFKGKATLTADKSSSTAYMQLSSLTSEDSAVYYCARSTYYGGDWYFNVWGAGTTVTVS 120
                  ************************************************************

Rituximab_HC      AASTKGPSVFPLAPSSKSTSGGTAALGCLVKDYFPEPVTVSWNSGALTSGVHTFPAVLQS 180
constructed       AASTKGPSVFPLAPSSKSTS-NTAALGCLVKDYFPEPVTVSWNSGALTSGVHTFPAVLQS 179
                  ******************** .**************************************

Rituximab_HC      SGLYSLSSVVTVPSSSLGTQTYICNVNHKPSNTKVDKKAEPKSCDKTHTCPPCPAPELLG 240
constructed       SGLYSLSSVVTVPSSSLGTQTYICNVNHKPSNTKVDKKAEPKSCDKTHTCPPCPAPELLG 239
                  ************************************************************

Rituximab_HC      GPSVFLFPPKPKDTLMISRTPEVTCVVVDVSHEDPEVKFNWYVDGVEVHNAKTKPREEQY 300
constructed       GPSVFLFPPKPKDTLMISRTPEVTCVVVDVSHEDPEVKFNWYVDGVEVHNAKTKPREEQY 299
                  ************************************************************

Rituximab_HC      NSTYRVVSVLTVLHQDWLNGKEYKCKVSNKALPAPIEKTISKAKGQPREPQVYTLPPSRD 360
constructed       NSTYRVVSVLTVLHQDWLNGKEYKCKVSNKALPAPIEKTISKAKGQPREPQVYTLPPSRD 359
                  ************************************************************

Rituximab_HC      ELTKNQVSLTCLVKGFYPSDIAVEWESNGQPENNYKTTPPVLDSDGSFFLYSKLTVDKSR 420
constructed       ELTKNQVSLTCLVKGFYPSDIAVEWESNGQPENNYKTTPPVLDSDGSFFLYSKLTVDKSR 419
                  ************************************************************

Rituximab_HC      WQQGNVFSCSVMHEALHNHYTQKSLSLSPG 450
constructed       WQQGNVFSCSVMHEALHNHYTQKSLSLSPG 449
                  ******************************
```

S9.3 Case study for I/L difference in BEV LC

Sequence alignment shows a difference of Ile and Leu in BEV light chain position 29. The ground-truth sequence indicates that it should be Ile, while our sequenced result shows Leu (with low confidence). From the statistics of our sequencing results, we found one PSM with a w ion to support the identification for L, and no PSM with a w ion to support that for I. Below is the PSM annotation and ion match table to support L.

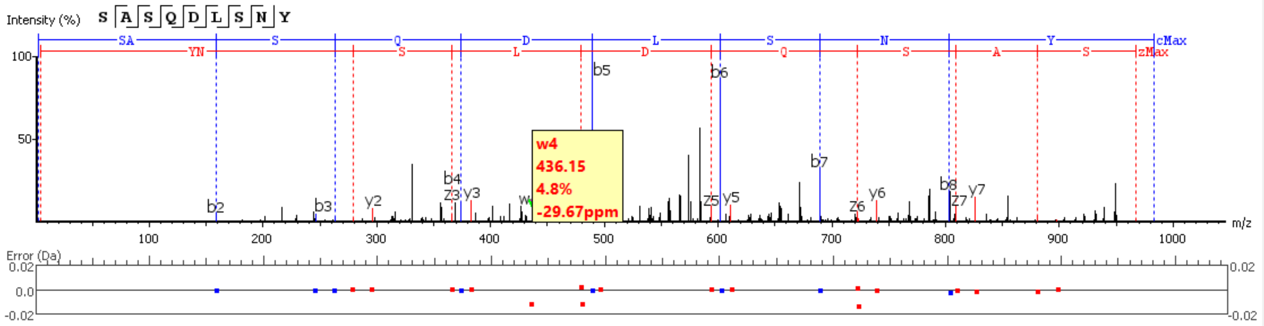

(a) PSM with w ion to support Leu. File: 20240325_4-anti_Chymo_ETHCD_SH_Ec.raw; Scan number 3639.

#	b	c	c-H	Seq	y	z	z'	w	w'	#
1	88.04	105.07	104.06	S						9
2	159.08	176.10	175.10	A	897.39	880.37	881.38			8
3	246.11	263.13	262.13	S	826.36	809.33	810.34			7
4	374.17	391.19	390.19	Q	739.32	722.30	723.29			6
5	489.19	506.22	505.21	D	611.27	594.24	595.25			5
6	602.28	619.31	618.30	L	496.24	479.22	480.21	436.15	437.17	4
7	689.31	706.34	705.33	S	383.16	366.13	367.14			3
8	803.35	820.38	819.37	N	296.12	279.10	280.10			2
9				Y	182.08	165.05	166.06			1

(b) Ion match table with w ion to support Leu. Coloured in red or blue means matched to a peak in the spectrum.

Figure S9.1: Spectrum support for BEV LC position 29 to be Leu.

S10 Experiment with Different Mixture Ratios

S10.1 Sample Preparation, Mass Spectrometry Instrumentation and Data Collection

USP1, USP2, and NIST antibodies were mixed in 3:3:1, 5:5:1, and 10:10:1 ratios, respectively, with a final amount of 20 μg per mixture. The mixtures were deglycosylated by adding 0.2 μl PNGase F (NEB, 15000U, P0705S) at 37°C, incubated overnight. Half of each mixture was reduced by adding 25 mM TCEP and incubating at 37°Cfor 30 mins. Each sample was dried and resuspended in 10 μl of 0.1% formic acid prior to MS analysis.

Deglycosylated (non-reduced) samples were run on a Thermo Scientific Orbitrap Fusion Lumos, equipped with a heated electrospray ionization source (H-ESI) in positive ion mode with a Thermo Fisher Ultimate 3000 RSLCnano HPLC System. On H-ESI source, sheath gas was set to 10 arbitrary units (arb), and auxiliary gas was set to 10 arb. The ion transfer tube was set at 325°C. The vaporizer temperature was at 350°C. The sample was separated on a MAbPac RP, 4μM, 3.0×100 mm analytical column, held at 60°C. The protein was eluted at a rate of 500 μL/min for a 10-minute gradient, where 0-7 minutes: 10% - 70% acetonitrile + 0.1% formic acid; 7-8.2 minutes: 95% acetonitrile + 0.1% formic acid, 8.2-10 minutes: 20% acetonitrile + 0.1% formic acid. MS spectra were acquired using full scans at 15000 resolution in the orbitrap within a scan range of 1500-3500 m/z. The maximum injection time was set at auto with a standard AGC target. Ten micro scans were employed, and the RF lens was set to 100%. 15V of insource CID was applied.

Deglycosylated and reduced samples were run on a Thermo Scientific Orbitrap Exploris 240 mass spectrometer, equipped with a heated electrospray ionization source (H-ESI) in positive ion mode with a Thermo Fisher Ultimate 3000 RSLCnano HPLC System. On H-ESI source, sheath gas was set to 10 arbitrary units (arb), and auxiliary gas was set to 10 arb. The ion transfer tube was set at 325°Cand the vaporizer temperature was at 350°C. The sample was separated on a MAbPac RP, 4μM, 3.0×100 mm analytical column, held at 60°C. The protein was eluted at a rate of 500 μL/min for a 10-minute gradient, where 0-7 minutes: 10% - 70% acetonitrile + 0.1% formic acid; 7-8.2 minutes: 95% acetonitrile + 0.1% formic acid, 8.2-10 minutes: 20% acetonitrile + 0.1% formic acid. MS spectra were acquired using full scans at 15000 resolution in the orbitrap within a scan range of 700-2200 m/z. The maximum injection time was set at auto with a standard AGC target. Ten micro scans were employed, and the RF lens was set to 100%. 15V of insource CID was applied.

S10.2 LC-MS Intact Analysis Results

From Figures S10.1 and S10.2, differing abundances of the NIST mAb and corresponding light and heavy chains are observed. Specifically, for the ratio of 10:10:1 (USP1:USP2:NIST), the deglycosylated whole NIST mAb is no longer observable in LC-MS. Our automated algorithm will not sequence an antibody if it is not mass-observable. Therefore, we know the limitations of our software's sensitivity. However, different molecules have varying ionization efficiencies, as observed in our four mAb experiment as well as this ratios experiment. The exact ratio limit at which our software fails to sequence is dependent on the sample.

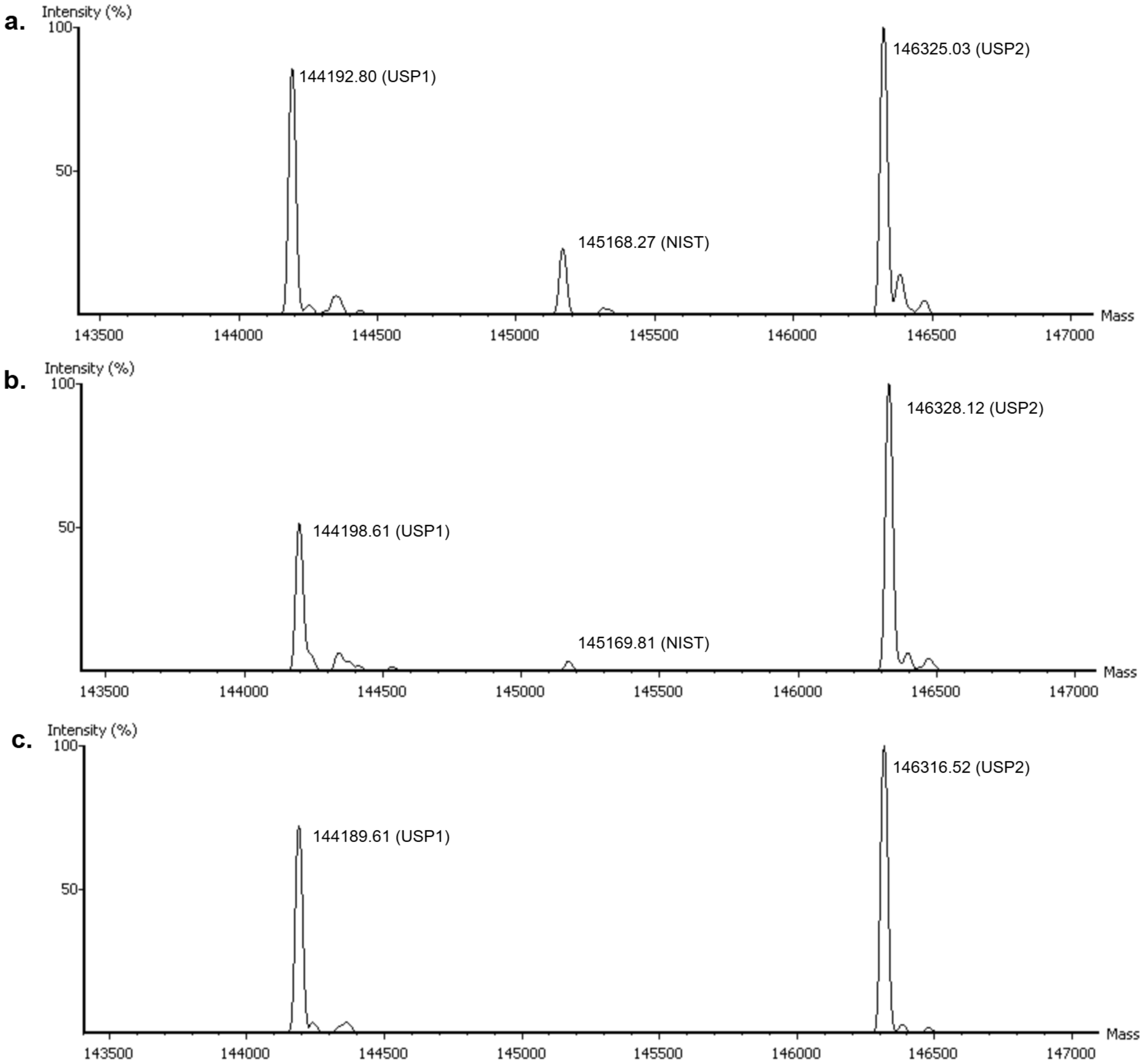

Figure S10.1: Deconvoluted Intact Mass Results for deglycosylated USP1, USP2, and NIST mAb mixture with ratios **a.** 3:3:1, **b.** 5:5:1, and **c.** 10:10:1. When the ratio is 10:10:1, the NIST mAb is no longer observable in LC-MS.

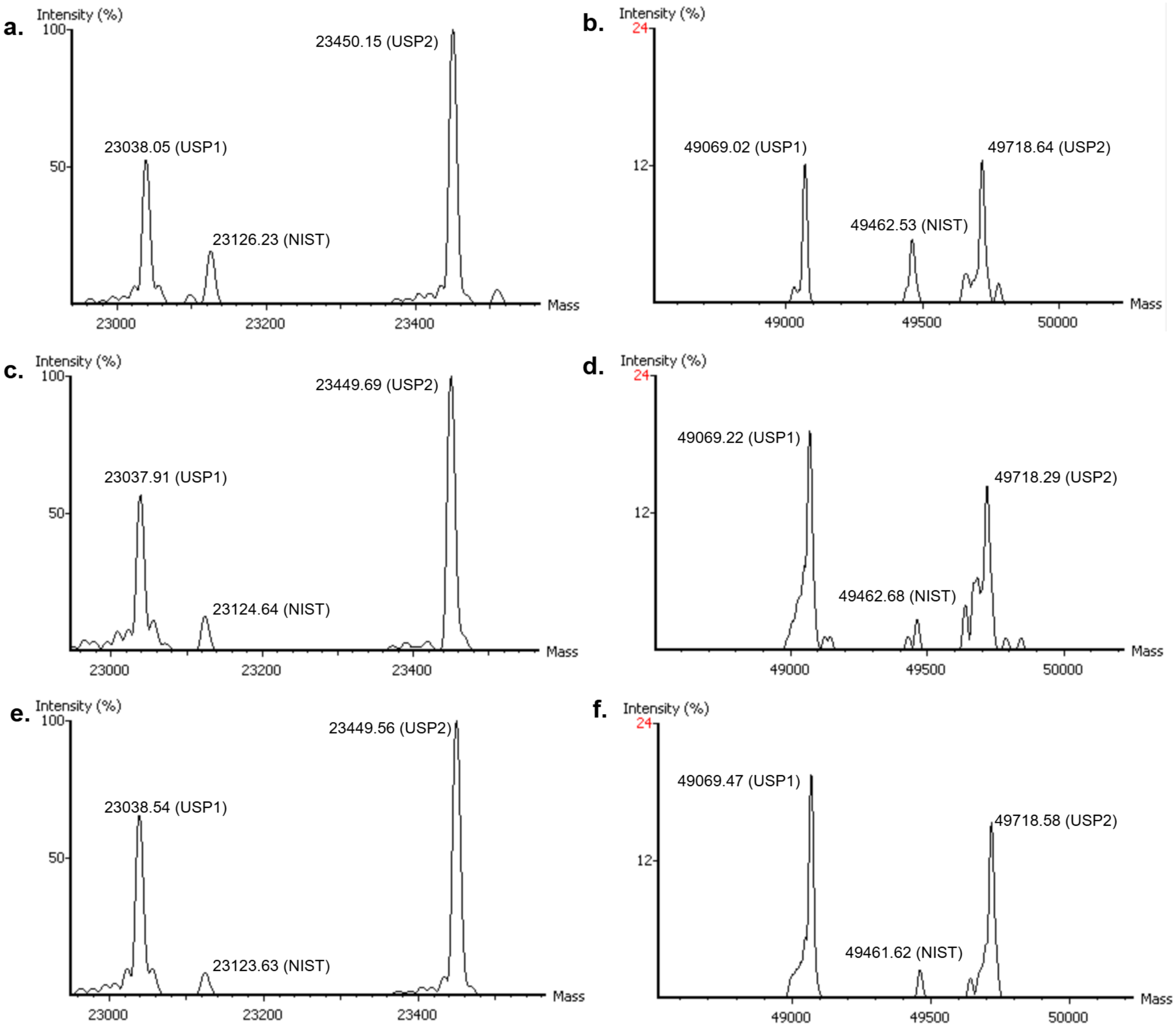

Figure S10.2: Deconvoluted Intact Mass Results for deglycosylated and reduced USP1, USP2, and NIST mAb mixture with different ratios: 3:3:1 (**a.** for LC and **b.** for HC), 5:5:1 (**c.** for LC and **d.** for HC), and 10:10:1 (**e.** for LC and **f.** for HC). Differences in ionization efficiencies are observed for different molecules.

S11 Additional LC-MS Examples

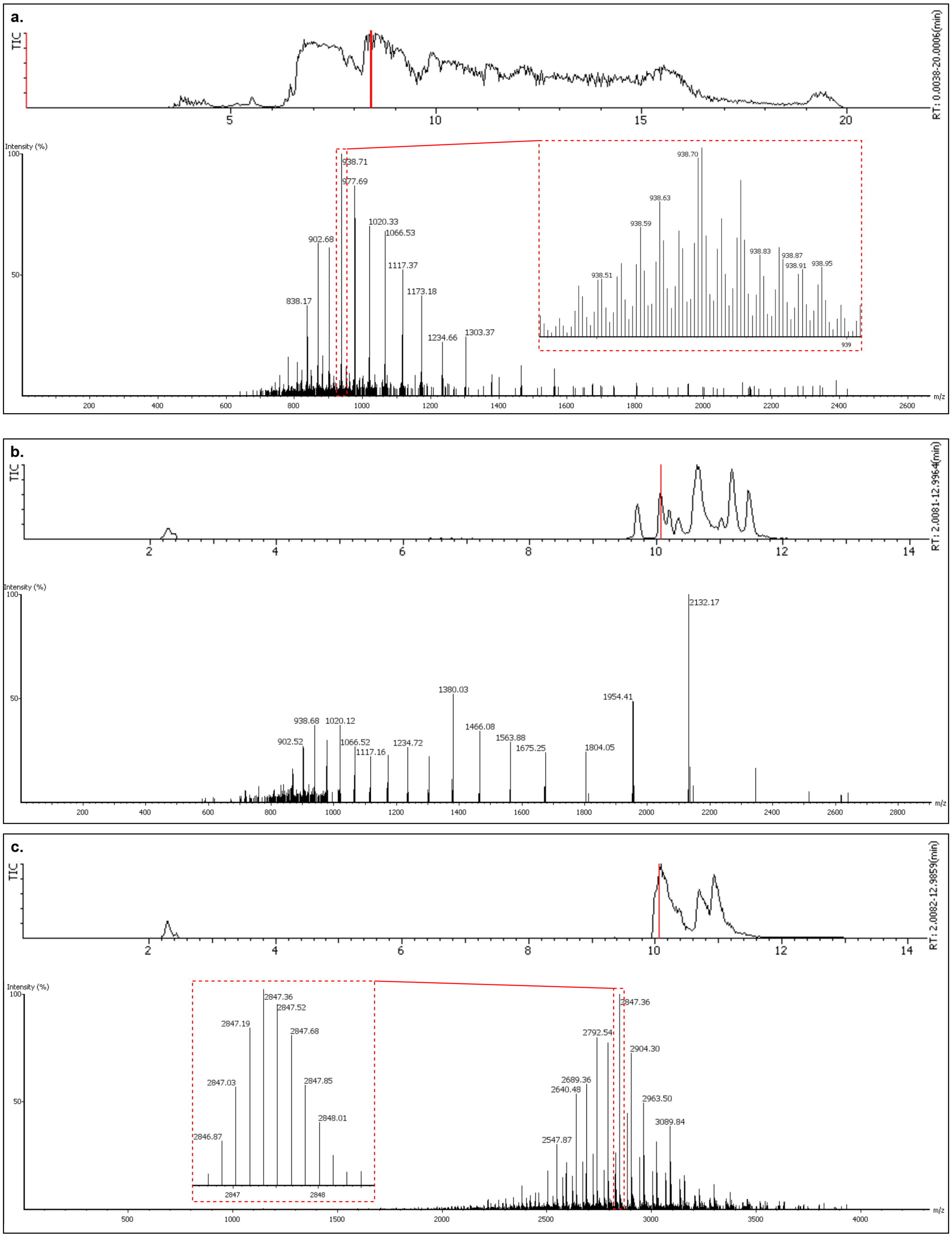

Figure S11.1: TIC and select MS1 raw spectra for 4 mAb intact for **a.** subunit, **b.** reduced, and **c.** whole samples.

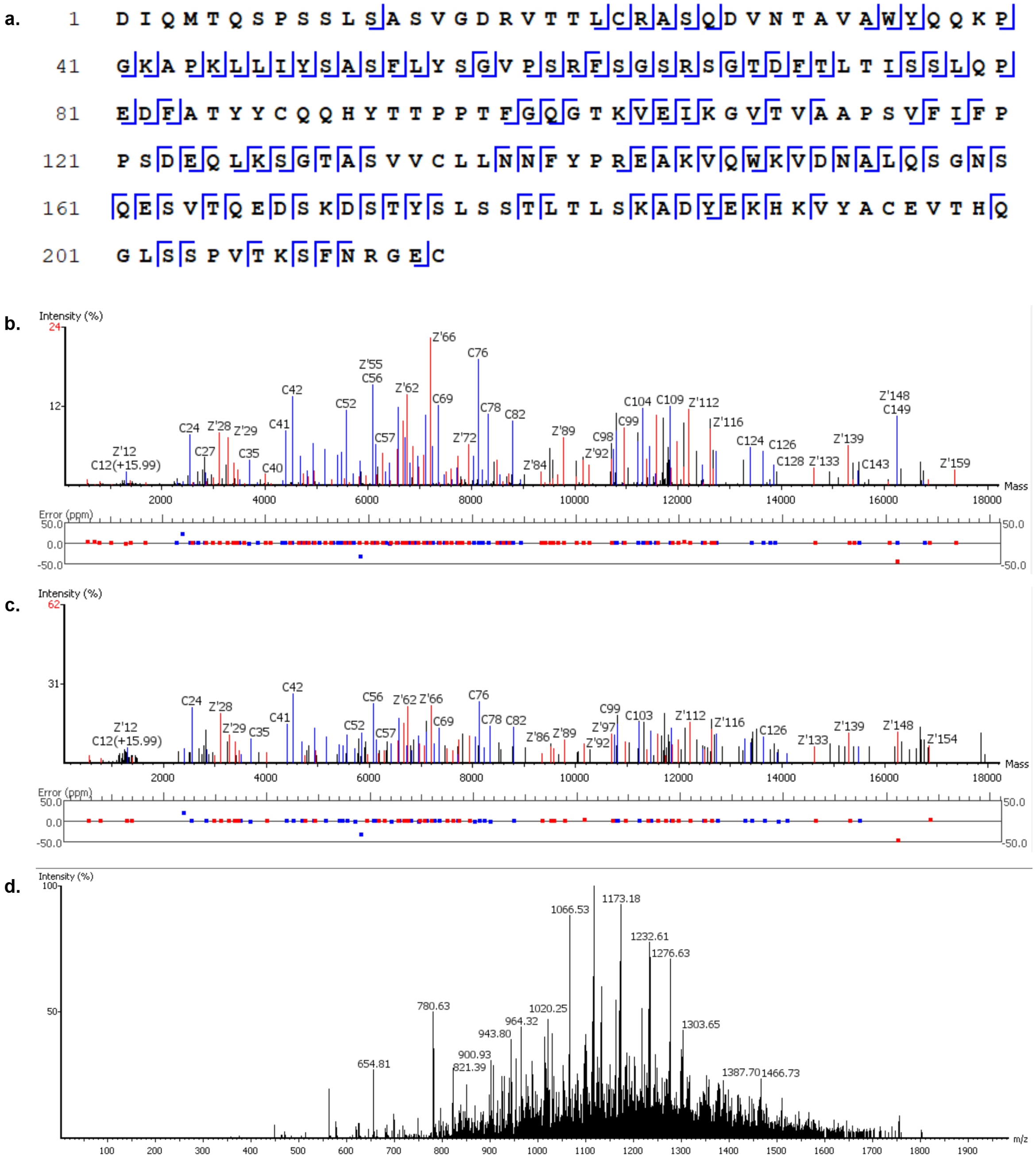

Figure S11.2: **a.** Middle-down matches for TRA LC with coverage 58.60%. **b.** annotated fragment ion matches of TRA LC for the merged deconvoluted spectrum. Middle-down MS2 spectra associated to the same intact mass are merged after deconvolution. **c.** annotated fragment ion matches of TRA LC for one deconvoluted spectrum from file "20240528_PA_SH000_ETD3MS_center_ABlist_1.raw" scan number 29. **d.** raw MS2 spectrum which generated the deconvoluted spectrum in (c.).
